# Pandemic or panic? A firm-level study on the psychological and industrial impacts of COVID-19 on the Chinese stock market

**DOI:** 10.1186/s40854-022-00335-8

**Published:** 2022-03-01

**Authors:** Qiuyun Wang, Lu Liu

**Affiliations:** School of Economics, Southwestern University of Finance and Economics, 555 Liutai Avenue, Wenjiang District, Chengdu, 611130 Sichuan China

**Keywords:** Black swan event, COVID-19, Psychological and industrial impacts, Shocks, Stock market reaction, G14, H12, I18, O16

## Abstract

This study presents a thorough investigation of the relationship between the coronavirus disease 2019 (COVID-19) and daily stock price changes. We use several types of COVID-19 patients as indicators for exploring whether stock prices are significantly affected by COVID-19’s impact. In addition, using the Chinese stock market as an example, we are particularly interested in the psychological and industrial impacts of COVID-19 on the financial market. This study makes two contributions to the literature. First, from a theoretical perspective, it shows a novel quantitative relationship between the psychological response to the pandemic and stock prices. In addition, it depicts the mechanism of the shock to the stock market by pointing out the specific functional expression of the impulse reaction. To our knowledge, this is the first theoretical calculation of the impulse of a shock to the financial market. Second, this study empirically estimates the marginal effect of the COVID-19 pandemic on fluctuations in stock market returns. By controlling for stock fundamentals, this study also estimates diverse industrial responses to pandemic stock volatility. We confirm that the COVID-19 pandemic has caused panic in the stock market, which not only depresses stock prices but also inflates volatility in daily returns. Regarding the impulse of the shock, we identify the cumulative level of the pandemic variables as well as their incremental differences. As shown by our empirical results, the terms for these differences will eventually dominate the marginal effect, which confirms the fading impulse of the shock. Finally, this study highlights some important policy implications of stock market volatility and returns to work in the industry.

## Introduction

On November 26, 2021, the newly emerged variant of SARS-CoV-2 (B.1.1.529), which was formally named by the World Health Organization (WHO) as “Omicron,” shocked the global stock markets. It was supposed to be the “Black Friday” for shopping in the U.S. and many other places. Instead, it became a real “Black Friday,” as panic caused a global stock market collapse.

This “Black Friday” reminds us of the crash of the American stock market beginning on February 26, 2020, as the daily rates of infection of coronavirus disease 2019 (COVID-19) began to increase in many regions around the globe. In February 2020, as the number of COVID-19 infections began to rise in many regions around the globe, the US stock market indices dropped sharply, and on June 26, 2020, this scenario was repeated, corresponding to a recent spike in infection rates in the US and elsewhere. This close relationship between stock market performance and evolution in the COVID-19 pandemic has attracted attention in academia and industry, as well as from social policymakers.

In recent decades, several pandemics (e.g., H1N1, SARS, and Ebola) have broken out, but none with the far-reaching, global, and colossal impact of COVID-19. Therefore, an analysis of the economic and financial impacts of COVID-19 makes for a unique contribution in understanding their intrinsic mechanisms as well as the complex relationship. In addition, because China has mostly contained the COVID-19 pandemic, whereas in many other regions it continues to spread widely, using the Chinese stock market as a research sample for studying the shock to the financial market from the pandemic can yield illuminating results for stock markets in other regions.

In December 2019, Wuhan became the epicenter of an outbreak of viral pneumonia in China, which was later formally called COVID-19 by the WHO. To prevent the further spread of infection, on January 23, 2020, the Chinese government implemented isolation measures in several provinces and cities, such as Beijing and Shanghai, in addition to Wuhan. The WHO subsequently classified the event as a public health emergency of international concern.^1^ The rapid spread of COVID-19 has already threatened hundreds of thousands of residents and has damaged the economy in China and the rest of the world. Restaurants are closed, and enterprises have reduced their operations. Moreover, the growing public concern over the breadth of the pandemic quickly spread to the stock market, with negative effects. For example, in China, on February 3, 2020, the opening day after the national holiday of the 2020 Spring Festival, the pandemic led to a drop of 7.7% in the Shanghai Composite Index and 8.5% in the Shenzhen Composite Index. To address this, the China Securities Regulatory Commission (CSRC) suspended all sales of securities.^2^

In February, as COVID-19 continued to evolve, the Chinese government launched several preventive and control measures. However, delaying the return to work and increasing traffic control in China may have periodic impacts on economic growth. For example, many industries in consumer retail and transportation are significantly affected by the pandemic. According to a report quoted by Standard and Poor's global rating, if consumer spending falls by 10%, China's overall gross domestic product (GDP) growth will fall by approximately 1.2%.^3^ On the one hand, every year, the Spring Festival (i.e., Chinese New Year) involves the largest population movement in China, which could accelerate the spread of COVID-19, making it more difficult to control. On the other hand, the Spring Festival is also associated with the year’s peak consumption. However, in 2020, the pandemic hit the service industries severely, as they were preparing for tourists at hotels, transportation, catering, entertainment, retail, and so on. All of these industries suffered significant losses.

The economic impact of COVID-19 is directly and indirectly affected by restrictions on population flow. According to data released by the Ministry of Transportation of the People’s Republic of China, the overall transportation volume dropped 28.8% on the first day of the Chinese lunar new year in 2020 compared to the prior year.^4^ The number of people traveling during the Spring Festival also declined dramatically: 415 million in 2019, but only 152 million in 2020.^5^ The Chinese government halted domestic group tourism, which closed nearly 20% of domestic routes because of the sharp drop in the number of passengers. In addition, domestic consumption, one of the most important driving forces of China's economy, inevitably experienced huge losses. According to the China Cuisine Association, during the Spring Festival, 78% of the catering enterprises lost 100% of their revenue in 2020 compared with 2019. In the first quarter of 2019, turnover in the accommodations and catering industry was RMB 423.4 billion but, for the first quarter of 2020, it was expected to lose about RMB 210 billion.^6^

China’s stock market is thus strongly affected. On the first trading day after the Spring Festival in 2020, the Shanghai stock index dropped by 8.5%, with more than 3,000 stocks falling. Similarly, in March 2003, when infections with SARS-CoV surged, the Stock Exchange of Hong Kong fell by about 10%^7^ and sent the MSCI China index tumbling by 8.6%. It increased by 14.7% in a month and 30.9% over three months.^8^ In 2016, the Zika virus spread in Brazil, and the MSCI Brazil index fell by approximately 3%. However, it increased by 14.8% after one month and 35.4% after three months.^9^ In 2018, the Ebola virus spread in the Congo, and the MSCI World Index fell by 7% in one month.^10^ A look at similar events and trends in history indicates that the impact of major pandemics on the stock market is complex. A comparison of COVID-19 and SARS shows totally different economic cycles and external environments. Around 2003, the trade growth of China and the world was very strong. In 2001, after China's accession to the World Trade Organization (WTO), the rate of total foreign trade growth increased from 21.8% in 2002 to 37.1% in 2003 but decreased from 9.6% in 2018 to 3.4% in 2019. Therefore, after the SARS epidemic in 2003, the economy recovered quickly.^11^ In 2003, China's investment and industrial growth fell briefly in the second quarter, followed by an upward trend. The rebound in trade and industry was very strong.^12^ The real estate suffered a clear decline, but it rebounded strongly afterward. Nevertheless, according to data from China's National Bureau of Statistics, the SARS epidemic reduced China's GDP growth rate by 0.8% in 2003.^13^

In recent years, “black swan” events have occurred more frequently. Each leads to an immediate reaction to international stock markets, foreign exchange markets, and various commodity markets. For instance, on June 24, 2016, Britain held a referendum on membership to the European Union, and those who supported leaving the EU won, with 51.9% of the vote. After the results were announced, the British pound dropped more than 10% in a single day, plunging to its lowest level in 31 years.^14^ On December 5, 2016, a referendum on constitutional amendments was held in Italy, and it was rejected. leading the country’s prime minister, Matteo Renzi, to announce his resignation. That day, the euro fell 1.4%, to its lowest level since March 2015, and the Italian 10-year bond yield exceeded 2% for the first time.^15^

Over the past few decades, China has experienced several black swan events in the financial market. Crises over “toxic infant formula [milk powder],” “toxic capsules,” “ineffective vaccines” and financial fraud produced significant losses for their investors in the stock market, as well as the corresponding food and drug industries. Although these events led to many short-term fluctuations in the financial market, many domestic investors are still unaware of how to identify and deal with uncertainty under certain market conditions. Black swan events lead to public panic. However, if the public overreacts to these events, the entire capital market suffers. Thus, appropriate policy interventions are needed, such as policies to prevent liquidity problems in the market. To address the negative impacts of the COVID-19 pandemic, many enterprises have accelerated product innovation and changes in their business models and organizational management models. Corporate social responsibility (CSR) has also been studied (Bae et al. 2021). In addition, both the central and local governments have actively introduced various measures to stabilize growth, which are expected to compensate for the loss of economic growth caused by the pandemic.

Although various types of economic shocks, particularly to stock markets, are familiar to us, the COVID-19 pandemic, which has huge and far-reaching impacts, has introduced several new challenges. As we have seen, different industries behave diversely in response to the pandemic, and the mechanism of the transmission of these shocks remains a mystery. When this mechanism is combined with psychological impacts, the relationship is much more difficult to comprehend. Undoubtedly, the COVID-19 pandemic is a disaster, and it requires new insights to understand many things that we thought were already largely known. Therefore, this study digs deeper into the specific mechanisms through which the pandemic affects stock prices and volatility.

Will the psychological issues arising from COVID-19 affect the stock market? Currently, we are not certain. Will the news about medical workers’ fight against COVID-19 as well as the rate of recovery or deaths affect the performance of the stock market? We do not know this either. Notably, along with quarantine activities, does the number of suspected COVID-19 infections affect investor behavior in the stock market? Currently, there is scant evidence in the literature to answer this question.

Using China as an example, this study conducts a deeper analysis of the impact of the COVID-19 outbreak on the stock market. Everything is intertwined during that period, so this is a very challenging problem to tackle. Therefore, we must isolate the impact of the COVID-19 pandemic from other possible influential factors. This study thoroughly investigates the relationship between COVID-19 and changes in daily stock prices. We use various types of COVID-19 patients as indicators to explore whether stock prices are significantly affected by COVID-19. In addition, we are particularly interested in the psychological and industrial impacts of COVID-19 on the financial market using samples from the Chinese stock market.

This study makes two contributions to the literature. First, it contributes theoretically in the sense that it shows a novel quantitative relationship between the psychological response to a pandemic and stock prices, perhaps the first study to do so. In addition, it depicts the mechanism of a shock to the stock market by pointing out the specific functional expression of the impulse reaction. To our knowledge, this might also be the first time that the impulse of a shock to the financial market has been calculated from a theoretical perspective. Second, this study empirically estimates the marginal effect of the COVID-19 pandemic on fluctuations in stock market returns. By controlling for stock fundamentals, this study estimates the effect of diverse industrial responses to the pandemic on stock volatility as well. Finally, this study has important policy implications regarding stock market volatility and the resumption of industrial work.

The paper is structured as follows. A literature review is presented in Sect. 2. Section 3 describes a novel theoretical framework that links the psychological and industrial impacts of COVID-19. Section 4 presents the study’s data. Section 5 presents the empirical arguments with the factors used as variables that might affect the daily returns on stock market prices, according to the theory. Section 6 discusses several important issues regarding empirical methods and corresponding results. Finally, the conclusions are presented in Sect. 7.

## Literature review

### Shocks to the stock market

The literature on stock market fluctuations is extensive (Barsky and Long 1993; Barlevy and Veronesi 2003; Engle et al. 2013). One of the most important driving forces in fluctuations is the shock to the stock market. The literature describes various types of shocks to the economy, including aggregate shocks (Hahn et al. 2020), shocks to exports (Amiti and Weinstein 2011; Caliendo et al. 2019), commodity prices (Hastings and Shapiro 2013), labor supply (Dustmann et al. 2017; Kim et al. 2018; Kong and Prinz 2020), dual-earner couples (Crawford et al. 2019), personal financial wealth (Bleakley and Ferrie 2016), and other financial markets, such as those for exchange rates (Eichenbaum and Evans 1995). Moreover, this strand of study also covers information shocks (Hung et al. 2015; Berger et al. 2020). Even investment bankers’ careers are linked to shocks to the stock market (Oyer 2008).

### Black Swan events and the financial market

Concerning the shock to the financial market associated with black swan events, some scholars find that uncertainty shocks cause fluctuations in consumption, investment, productivity, and stock market volatility (Beaudry and Portier 2004; Berger et al. 2015; Basu and Bundick 2017). Reactions to monetary and fiscal policies regarding the stock market have also been discussed (Hassett and Metcalf 1999; Mueller 2001; Rigobon and Sack 2003; Christiano et al. 2005; Fratzscher and Rieth 2019). In addition, stock market reactions to political shocks are also significant factors (Kaustia and Torstila 2011; Wagner et al. 2018). The existence of a wealth shock to the stock market cannot be overlooked (Gormley et al. 2010). In addition, both investment shocks (Papanikolaou 2011) and credit shocks (Khan and Thomas 2013) were found to be important. Moreover, Forbes and Rigonbon (2002) show that financial contagion leads to a significant increase in cross-market linkages after a shock or financial crisis in a country. Finally, social movements are also found to affect stock price returns (King and Soule 2007).

To further investigate the stock market’s reaction to black swan events, studies explore the impact of these events, which are composed of economic events, social events, acts of terrorism, and natural disasters.

To begin with, studies have investigated the reaction of stocks to major international terrorist events, and they have found that most of them have mildly positive or negative effects on stocks in the long run. Although significant effects are found in the short run, the stock market recovers quickly. The only event with a significant effect was the attack on 9/11 (Nikkinen et al. 2008; Brounrn and Derwall 2010; Hanabusa 2010; Liargovas and Repousis 2010; Zopiatis et al. 2017). Karolyi and Martell (2005) indicate that attacks in more advanced countries are associated with larger negative share price reactions.

Moreover, scholars have also discussed the impact of natural disasters on stock markets. Baker and Bloom (2013) investigate the impact of natural disasters, terrorist attacks, and unexpected political shocks on economic growth. They find that the impact is the largest in countries with less developed financial markets and stiffer labor markets. Some researchers find no significant impact on market returns (Worthington 2008) or negative returns only on the day of the event (Caporale et al. 2019). Other scholars find negative stock price reactions to natural disasters, which cause great damage to the economy (Yamori and Kobayashi 2002; Wang and Kutan 2013; Tavor and Teitler-Regev 2019). Worthington and Valadkhani (2004) analyzed different genres of natural disasters and found that different kinds of natural disasters have different impacts on the Australian equity market.

### Other common types of influential shocks

Furthermore, major world news is also an influential factor in stock markets. Scholars who have analyzed stock market reactions to world news believe that major news about wars, politics, financial policies and sudden public scandals affect stock prices (Niederhoffer 1971; Zhou and Zhao 2013). The difference in total stock returns can be attributed to various types of news (Cutler et al. 1989). Stock prices may also depend on the stock trading activity. Robinson and Bangwayo-Skeete (2017) show that stock prices in markets that are less active do not react to the vast majority of major news events. In this strand of the literature, major events in the stock market, such as a link between poison pills and stock market reactions, have been studied (Rhee and Fiss 2014; Dorobantu et al. 2017).

Energy accidents that cause fluctuations in the stock market have also been considered. It appears that the stock market, in general, did not show a significant reaction to major energy accidents from 1907 to 2007 (Sovacool et al. 2008). Scholtens and Boersen (2011) support Sovacool et al., who conclude that stock prices did not react significantly to environmental accidents at energy firms that occurred between 1907 and 2007.

### The shock of COVID-19 to the economy and the financial markets

The COVID-19 pandemic has evolved very quickly, and so has the academic understanding of its complex impact on the economy as well as financial markets.

Now, the terminology of “COVID-19 shock” has been formally proposed in the financial academia (Caballero and Simsek 2021). The pandemic has triggered shocks to technology, finance, the economy, and government policy (Gu et al. 2020; Haroon and Rizvi 2020; Tisdell 2020; Zaremba et al. 2020; Sharma et al. 2021), often accompanied by capital underutilization, manufacturing output decline, production cost inflation, and a decrease in demand for certain services. Satif et al. (2021) described the possible impact of COVID-19 on bilateral trade. A negative trend is observed in the supply of and demand for labor, which harms the service sector, leading to further discussion about the security of all sectors and the prevention of unemployment (Ceylan et al. 2020).

Observing the relationship between oil prices and stock returns also allows us to see whether the COVID-19 pandemic has changed. The implications of the impacts on financial markets, that is, stock returns, of the pandemic have provided preliminary insights. Inspired by the cash-flow hypothesis, the increase in production cost leads to an increase in oil prices; therefore, the dividends of cash flows decline and, as a result, stock returns. Zhang et al. (2021) demonstrate that, because of COVID-19, the influence of oil prices on stock returns decreased by approximately 89.5%.

Researchers have studied the impact of COVID-19 on financial markets from several perspectives (Gu et al. 2020; Heyden and Heyden 2020; Apostolakis et al. 2021; Liu et al. 2021; Nigmonov and Shams 2021). Existing literature shows that COVID-19 affects the stock market. Some find that the total number of infections and deaths have negative effects on stock market returns (Al-Awadhi et al. 2020; Ashraf 2020a; Zhang et al. 2021) and a significant long-term impact on the most affected countries (Sharma et al. 2021). However, the evidence shows that the impact on the Chinese stock market is short-lived because of the implementation of government policies (Hu et al. 2020). The short-term reactions of the stock market around the world during the COVID-19 pandemic have also been examined (Heyden and Heyden 2020; Rahman et al. 2020). COVID-19 hurts solar energy stock prices in both the short and long run, but the effects are not significant in non-OECD countries (Wei et al. 2021).

Theoretically, investors are likely to overreact and adopt a conservative approach to investment decisions because of COVID-19 (Shear et al. 2020; Aslam et al. 2021). Huber et al. (2021) indicate that higher risk aversion during a pandemic might reduce investment, even though experimental assets are less risky. However, Angrisani et al. (2020) believe that the increase in risk premia during the pandemic is due to a change in beliefs, but not due to changes in market participants’ appetite for risk.

As the disease has caused a continuous decline around the world, some scholars have analyzed the feelings of panic and constructed a global fear index for COVID-19 to use as an indicator of investment decisions (Haroon and Rizvi 2020; Papadamou et al. 2020; Salisu and Akanni 2020; Liu et al. 2021). The continuous decline in the global market has a financial risk spillover effect that devastates the entire financial system through negative returns, increased uncertainty, and higher volatility (Ashraf 2020b; Goodell and Goutte 2020; Sharif et al. 2020; Li et al. 2021; Yang and Yang 2021). The spillover effect during the COVID-19 pandemic has been examined, including the spillover between financial technology stocks and other financial assets (Lan et al. 2021) and volatility spillovers among European stock markets (Aslam et al. 2021; Youssef et al. 2021).

## Research gap

Finally, the absence of management in the study of financial crises is abnormal (Starkey 2015). In fact, crisis management is an important part of management (Pearson 2010; Bundy et al. 2017), especially at a time of a global financial crisis (DesJardine et al. 2019). Here, we attempt to add to the existing scholarship on the issue.

To our knowledge, few studies have been conducted on shocks to the financial market due to a pandemic, and most of them are only empirical. Therefore, the theoretical mechanism of how and why a pandemic causes a shock to the stock market remains unclear. Therefore, this study contributes to the literature by revealing the hidden mechanism in shocks as well as its empirical application to the financial market using the Chinese stock market as an example. Unlike the extant literature on similar topics, this study performs detailed mathematical modeling to depict the impact mechanism of a shock from a pandemic to the stock market; a significant theoretical contribution. In addition, the empirical section of this study employs comprehensive examinations and regressions.

## Theoretical framework

Here, we consider three categories of influential factors. The first is the fundamentals, which include the macroeconomic situation as well as the basic operating status of listed companies. The macroeconomic situation reflects the overall operating performance of listed companies and determines the further development of listed companies. The macroeconomic situation is closely related to listed companies and their corresponding stock prices. The basic operating status of listed companies includes their financial condition, profitability, market share, and management system.

The second category comprises psychological factors, which are mainly reflected by changes in stock prices. If people feel panic, they have negative attitudes toward the stock market, and thus prices fall. However, if people find out that they have overreacted to the pandemic or recovery rates increase, then they will regain confidence about the stock market, and stock prices will rebound.

The third category is industry factors. From our perspective, industries have different influences on stock price changes. Theoretically, after the outbreak of COVID-19, stock prices in health-related industries have risen because medical supplies are urgently needed. By contrast, stock prices in industries related to entertainment fell because people went to movie theaters, clubs, and theme parks much less often.

Hence, we introduce the following equation:

1 $$P = f(Fund,Psy,Ind).$$

where *P* is the firm-level stock price, *f(·)* is the function whose details are still unknown, *Fund* is firm-level fundamentals, *Psy* is psychological factors, and *Ind* is industry factors. All these functions are assumed to be continuous, and twice differentiable.

Differentiating this function with respect to time yields:

2 $$\begin{aligned} \mathop P\limits^{ \bullet } = f^{\prime } (Fund,Psy,Ind)\mathop {Fund}\limits^{ \bullet } + f^{\prime } (Fund,Psy,Ind)\mathop {Psy}\limits^{ \bullet } + f^{\prime } (Fund,Psy,Ind)\mathop {Ind}\limits^{ \bullet } . \\ \end{aligned}$$

As our primary research interest is in the percentage change in firm-level stock prices, we divide by *P* on both sides of the equation and obtain:

3 $$\begin{aligned} G_{P} = \frac{{\mathop P\limits^{ \bullet } }}{P} = f^{\prime } (Fund,Psy,Ind)\frac{{\mathop {Fund}\limits^{ \bullet } }}{P} + f^{\prime } (Fund,Psy,Ind)\frac{{\mathop {Psy}\limits^{ \bullet } }}{P} + f^{\prime } (Fund,Psy,Ind)\frac{{\mathop {Ind}\limits^{ \bullet } }}{P}. \\ \end{aligned}$$

where *G*_*P*_ is the growth rate or the rate of change in firm-level stock prices.

The right-hand side of the equation shows that over a short period, firm-level fundamentals do not change substantially, nor are they reflected in publicly released reports on corporations. Therefore, for simplicity, we substitute $\mathop {Fund}\limits^{ \bullet } = 0$. However, for *Psy* and *Ind*, things are much more complicated. Based on facts during the pandemic, we assume the following:

4 $$Psy = Psy(Epi).$$

5 $$Ind = Ind(Epi).$$

where *Epi* represents the COVID-19 pandemic. If we differentiate these two equations with respect to time, we have:

6 $$\mathop {Psy}\limits^{ \bullet } = Psy^{\prime } (Epi)\mathop {Epi}\limits^{ \bullet } .$$

7 $$\mathop {Ind}\limits^{ \bullet } = Ind^{\prime } (Epi)\mathop {Epi}\limits^{ \bullet } .$$

Substituting Eqs. (6) and (7) into Eq. (3) and rearranging the terms, we have

8 $$\begin{aligned} G_{P} = \frac{{\mathop P\limits^{ \bullet } }}{P} = \frac{{f^{\prime } (Fund,Psy,Ind)}}{P}(Psy^{\prime } (Epi) + Ind^{\prime } (Epi))\mathop {Epi}\limits^{ \bullet } . \\ \end{aligned}$$

Now, we take the logarithm on both sides of the equation and obtain:

9 $$\begin{aligned} \ln \left( {\frac{{\mathop P\limits^{ \bullet } }}{P}} \right) = \ln (f^{\prime } (Fund,Psy,Ind)) - \ln (P) + \ln (Psy^{\prime } (Epi) + Ind^{\prime } (Epi)) + \ln (\mathop {Epi}\limits^{ \bullet } ). \\ \end{aligned}$$

As shown, this equation has very close empirical implications. However, because many of the related functional forms are still unknown, we cannot directly apply them to empirical analysis.

We further define $Y = \ln (\frac{{\mathop P\limits^{ \bullet } }}{P})$ and then differentiate *Y* with respect to $Ind^{\prime}(Epi)$, obtaining:

10 $$\frac{\partial Y}{{\partial Ind^{\prime } (Epi)}} = \frac{1}{{Psy^{\prime } (Epi) + Ind^{\prime } (Epi)}}.$$

This equation is the second-order derivative of *Y* with respect to *Ind*, thus obtaining the following relationship:

11 $$\begin{aligned} \int {\frac{\partial Y}{{\partial Ind^{\prime } (Epi)}}} dEpi = \frac{\partial Y}{{\partial Ind(Epi)}} = Ind^{\prime } (Epi) \\ \end{aligned}$$

Now, we integrate both sides of Eq. (10) and obtain:

12 $$\begin{aligned} \int {\frac{\partial Y}{{\partial Ind^{\prime } (Epi)}}} dEpi & = \int {\frac{1}{{Psy^{\prime } (Epi) + Ind^{\prime } (Epi)}}} dEpi \\ & = \frac{{\ln (Psy^{\prime } (Epi) + Ind^{\prime } (Epi))}}{{Psy^{\prime \prime } (Epi) + Ind^{\prime \prime } (Epi)}} + C_{1} . \\ \end{aligned}$$

where *C*_*1*_ is the integration constant.

By combining Eqs. (11) and (12), we obtain:

13 $$\frac{{\ln (Psy^{\prime } (Epi) + Ind^{\prime } (Epi))}}{{Psy^{\prime\prime}(Epi) + Ind^{\prime\prime}(Epi)}} + C_{1} = Ind^{\prime } (Epi).$$

After rearranging terms, we obtain:

14 $$\begin{aligned} \ln (Psy^{\prime } (Epi) + Ind^{\prime } (Epi)) = (Ind^{\prime } (Epi) - C_{1} )(Psy^{\prime\prime}(Epi) + Ind^{\prime\prime}(Epi)). \\ \end{aligned}$$

Now, we get to the “tricky” part. If we take the exponential form on both sides of Eq. (14), we obtain

15 $$\begin{aligned} Psy^{\prime } (Epi) + Ind^{\prime } (Epi) = \exp ((Ind^{\prime } (Epi) - C_{1} )(Psy^{\prime\prime}(Epi) + Ind^{\prime\prime}(Epi))). \\ \end{aligned}$$

Thus, we obtain:

16 $$\begin{aligned} Psy^{\prime } (Epi) = \exp ((Ind^{\prime } (Epi) - C_{1} )(Psy^{\prime\prime}(Epi) + Ind^{\prime\prime}(Epi))) - Ind^{\prime } (Epi). \\ \end{aligned}$$

To determine the key function of *Psy(Epi)*, we need to integrate Eq. (16) on both sides as follows:

17 $$\begin{aligned} \int {Psy^{\prime } (Epi)} dEpi & = \int {(\exp ((Ind^{\prime } (Epi) - C_{1} )(Psy^{\prime\prime}(Epi)} + Ind^{\prime\prime}(Epi))) - Ind^{\prime } (Epi))dEpi \\ & = \int {\exp ((Ind^{\prime } (Epi) - C_{1} )(Psy^{\prime\prime}(Epi)} + Ind^{\prime\prime}(Epi)))dEpi - \int {Ind^{\prime } (Epi)} dEpi \\ & = \int {\exp ((Ind^{\prime } (Epi) - C_{1} )(Psy^{\prime\prime}(Epi)} + Ind^{\prime\prime}(Epi)))dEpi - (Ind(Epi) + C_{2} ). \\ \end{aligned}$$

Focusing on the term $\int {\exp ((Ind^{\prime}(Epi) - C_{1} )(Psy^{\prime\prime}(Epi) + Ind^{\prime\prime}(Epi))} )dEpi$ and considering all the second-order terms constants for simplicity, we have

18 $$\begin{aligned} & \int {\exp ((Ind^{\prime}(Epi) - C_{1} )(Psy^{\prime\prime}(Epi) + Ind^{\prime\prime}(Epi)))} dEpi \\ & \quad = \frac{{\exp ((Ind^{\prime}(Epi) - C_{1} )(Psy^{\prime\prime}(Epi) + Ind^{\prime\prime}(Epi)))}}{{(Psy^{\prime\prime}(Epi) + Ind^{\prime\prime}(Epi))Ind^{\prime\prime}(Epi)}} + C_{3} . \\ \end{aligned}$$

where *C*_*2*_ and *C*_*3*_ are the integration constants in these steps. Substituting Eq. (18) into Eq. (17), we obtain:

19 $$\begin{aligned} & Psy(Epi) = \frac{{\exp ((Ind^{\prime}(Epi) - C_{1} )(Psy^{\prime\prime}(Epi) + Ind^{\prime\prime}(Epi)))}}{{(Psy^{\prime\prime}(Epi) + Ind^{\prime\prime}(Epi))Ind^{\prime\prime}(Epi)}} - Ind(Epi) + C_{4} . \\ \end{aligned}$$

where $$C_{4} = C_{3} - C_{2}$$.

After some complex derivations (see the mathematical appendix for details), we obtain:

20 $$\begin{aligned} Psy(Epi) + Ind(Epi) &= \exp (((Psy^{\prime\prime}(Epi) + Ind^{\prime\prime}(Epi))Ind^{\prime\prime}(Epi))Epi) \\&= (\exp (Epi))^{{((Psy^{\prime\prime}(Epi) + Ind^{\prime\prime}(Epi))Ind^{\prime\prime}(Epi))}} . \\ \end{aligned}$$

Although we have made a relatively strong assumption that all the second-order terms are constants, Eq. (20) offers a novel perspective for depicting the quantitative relationship between stock prices and the psychological response to a pandemic. If the specific functional forms of *Psy* and *Ind* are in quadratic form, then the assumption of constant second-order terms is reasonable, because the first-order condition of a typical quadratic function is linear.

By differentiating both sides of Eq. (20), we obtain

21 $$\begin{aligned} Psy^{\prime}(Epi) + Ind^{\prime}(Epi)& = ((Psy^{\prime\prime}(Epi) + Ind^{\prime\prime}(Epi))Ind^{\prime\prime}(Epi))\exp (((Psy^{\prime\prime}(Epi) \\ & \quad + Ind^{\prime\prime}(Epi))Ind^{\prime\prime}(Epi))Epi) = ((Psy^{\prime\prime}(Epi) \\ & \quad + Ind^{\prime\prime}(Epi))Ind^{\prime\prime}(Epi))(\exp (Epi))^{{((Psy^{\prime\prime}(Epi) + Ind^{\prime\prime}(Epi))Ind^{\prime\prime}(Epi))}} . \\ \end{aligned}$$

Then, we take the logarithm on both sides of Eq. (21), and we obtain

22 $$\begin{aligned} \ln (Psy^{\prime}(Epi) + Ind^{\prime}(Epi)) &= \ln (((Psy^{\prime\prime}(Epi) + Ind^{\prime\prime}(Epi))Ind^{\prime\prime}(Epi)) \\ & \quad + ((Psy^{\prime\prime}(Epi) + Ind^{\prime\prime}(Epi))Ind^{\prime\prime}(Epi))Epi. \\ \end{aligned}$$

Now, if we substitute Eq. (22) into Eq. (9), we obtain:

23 $$\begin{aligned} \ln \left( {\frac{{\mathop P\limits^{ \bullet } }}{P}} \right) & = \ln (f^{\prime}(Fund,Psy,Ind)) - \ln (P) + \ln (((Psy^{\prime\prime}(Epi) + Ind^{\prime\prime}(Epi))Ind^{\prime\prime}(Epi)) \\ & \quad + ((Psy^{\prime\prime}(Epi) + Ind^{\prime\prime}(Epi))Ind^{\prime\prime}(Epi))Epi + \ln (\mathop {Epi}\limits^{ \bullet } ). \\ \end{aligned}$$

Finally, by rearranging the terms, we obtain:

24 $$\begin{aligned} \ln \left( {\frac{{\mathop P\limits^{ \bullet } }}{P}} \right) & = \ln (f^{\prime}(Fund,Psy,Ind)((Psy^{\prime\prime}(Epi) + Ind^{\prime\prime}(Epi))Ind^{\prime\prime}(Epi)) \\ & \quad - \ln (P) + ((Psy^{\prime\prime}(Epi) + Ind^{\prime\prime}(Epi))Ind^{\prime\prime}(Epi))Epi + \ln (\mathop {Epi}\limits^{ \bullet } ). \\ \end{aligned}$$

The constant term in the regression is:

$\ln (f^{\prime}(Fund,Psy,Ind)((Psy^{\prime\prime}(Epi) + Ind^{\prime\prime}(Epi))Ind^{\prime\prime}(Epi))$, and the estimation coefficient for *Epi* is $((Psy^{\prime\prime}(Epi) + Ind^{\prime\prime}(Epi))Ind^{\prime\prime}(Epi))$, which is easy to understand and has a meaningful economic interpretation.

Moreover, to use the logarithm of real-world data in which some of the numerical values for the rate of change are negative, we multiply both sides of Eq. (24) by 2 and obtain:

25 $$\begin{aligned} 2\ln \left( {\frac{{\mathop P\limits^{ \bullet } }}{P}} \right) & = 2\ln (f^{\prime}(Fund,Psy,Ind)((Psy^{\prime\prime}(Epi)+ Ind^{\prime\prime}(Epi))Ind^{\prime\prime}(Epi)) \\ & \quad - 2\ln (P) + 2((Psy^{\prime\prime}(Epi) + Ind^{\prime\prime}(Epi))Ind^{\prime\prime}(Epi))Epi + 2\ln (\mathop {Epi}\limits^{ \bullet } ). \\ \end{aligned}$$

which is,

26 $$\begin{aligned} \ln \left( {\left( {\frac{{\mathop P\limits^{ \bullet } }}{P}} \right)^{2} } \right) & = 2\ln (f^{\prime}(Fund,Psy,Ind)((Psy^{\prime\prime}(Epi) + Ind^{\prime\prime}(Epi))Ind^{\prime\prime}(Epi)) \\ & \quad - \ln (P^{2} ) + 2((Psy^{\prime\prime}(Epi) + Ind^{\prime\prime}(Epi))Ind^{\prime\prime}(Epi))Epi + \ln ((\mathop {Epi}\limits^{ \bullet } )^{2} ). \\ \end{aligned}$$

Equation (26) suggests the combined use of the quadratic form of certain variables with the log form. This is the final expression that we obtain theoretically, which has meaningful practical empirical implications.

## Data

### The choice of time period

The use of daily data to study the volatility in the stock market has a long tradition (Turner and Weigel 1992). The period of our study was February 3–25, 2020. This period was selected for two reasons. First, Wuhan was officially sealed off on January 23, followed by the planned seven-day national holiday for the Chinese New Year, during which the stock market in China was closed but reopened on January 31. However, due to COVID-19, the reopening date was postponed to the following Monday, February 3, 2020. Second, as COVID-19 worsened outside China in late February, the US stock market fell on February 26, 2020, setting off a “chain reaction” first in global financial markets and later in China. Therefore, to isolate the outside interference to the Chinese stock markets, we stopped updating our data on February 25. As a result, the study period systematically reflects the local impact of COVID-19 on the Chinese stock market.

**Fig. 1:**
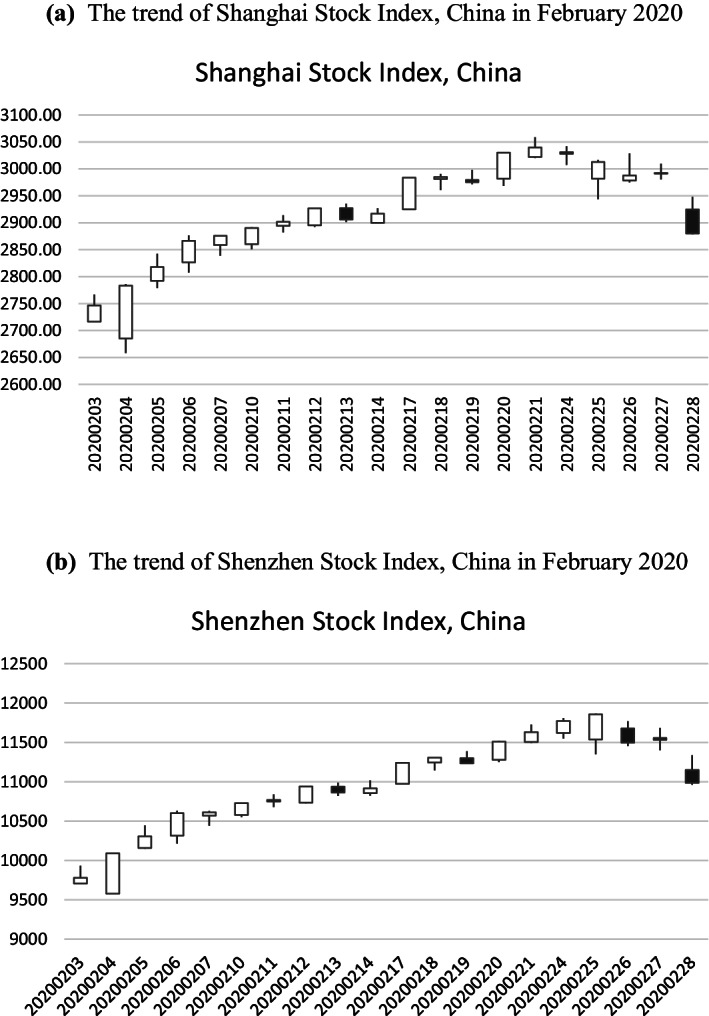
**a** The trend of Shanghai Stock Index, China in February 2020. **b** The trend of Shenzhen Stock Index, China in February 2020. *Source*: East Money

The additional reason that we select this period is that we are particularly interested in what happens after the “black swan” event mentioned in the introduction. After the Chinese stock market reopened, with a massive drop in value, on February 3, 2020, the stock prices of listed firms began to rise in some industries, continued to drop in others, and were neutral in other industries. As shown in Fig. 1a, b, after February 3, the stock market indices in China generally rose. After February 26, the stock market indices in Shanghai and Shenzhen both began to drop again, which confirms that the Chinese stock market is also affected by a crash in the US stock market. Therefore, the data after February 26 would be misleading.

The data set is structured as a panel that includes 3,759 individually listed companies as well as 16 trading dates. As mentioned earlier, we include the following three types of variables:

### COVID-19 patients

We extracted data on the pandemic from the National Health Commission of the People’s Republic of China (NHC),^16^ which comprises rates of infection, suspected infection, ICU patients, death, and recovery on a daily basis. Those who had a cough, fever, or breathlessness and had contact with someone infected with COVID-19 or returned from a high-risk area in the 14 days before the onset of symptoms were considered to have a suspected infection.

### Stock market variables

Our main purpose here is to discover the impact of COVID-19 shocks on the Chinese stock market, which means that the volatility of stock market variables is of great importance. In this study, research on the volatility of the stock market mainly focuses on changes in stock market prices, which is used as the main indicator to measure volatility in the stock market. Theoretically, stock market fluctuations consist of changes in stock prices caused by changes in a stock’s intrinsic value and external factors. Stock market prices change rapidly, but the basis of the stock price for listed companies is their financial performance; therefore, fluctuations in the stock market should be based on financial performance. Thus, as an important part of the stock market, listed companies’ financial performance is expected to have an important impact on stock market volatility. Based on the experience of mature markets, the performance of listed companies is the basis for the stable development of the stock market. If the overall performance of listed companies declines, the market foundation becomes unstable, which increases the risk and volatility of the entire market.

As we know, the intrinsic value of a stock is directly proportional to the earnings per share of the listed company, that is, if the company's operating performance is good, then the intrinsic value of its stock is correspondingly high; otherwise, it is low. Considering that the financial independence and complementarity of each index need to be displayed, we use total revenue, operating cost, and operating profit as proxies for the company’s market value, operating ability, profitability, and other aspects. The annual financial reports were obtained from the East Money database.^17^ Considering the practical value of the study and the validity of the data, we use the period covered by the financial report, which ended on December 31 in the year before the outbreak of COVID-19.

In addition, we use the buying volume, price-to-book ratio, total market capitalization, changes and percentage changes in daily returns as direct external factors in stock performance. The change in trading volume gives us important information about changes in market sentiment; hence, we use the buying volume to represent the market attitude. Furthermore, total market capitalization refers to how much a company is valued as determined by the stock market, and it is defined as the total market value of all outstanding shares. Using total market capitalization to show the size of a company is important because company size is a basic determinant of various characteristics in which investors are interested. Moreover, the price-to-book ratio (P/B) reflects the value that market participants place on a company’s equity relative to the book value of its equity. A stock’s market value is a forwarding-looking metric that reflects a company’s future cash flows. It is helpful to identify some general parameters or a range of P/B values, and then consider various other factors and valuation measures that more accurately interpret the P/B value and forecast a company’s potential for growth. Here, we gather stock shares listed on the Shanghai Stock Exchange (SHSE) and Shenzhen Stock Exchange (SZSE), which are the main stock exchanges in China. The data were also obtained from East Money.

### Industry variables

**Fig. 2:**
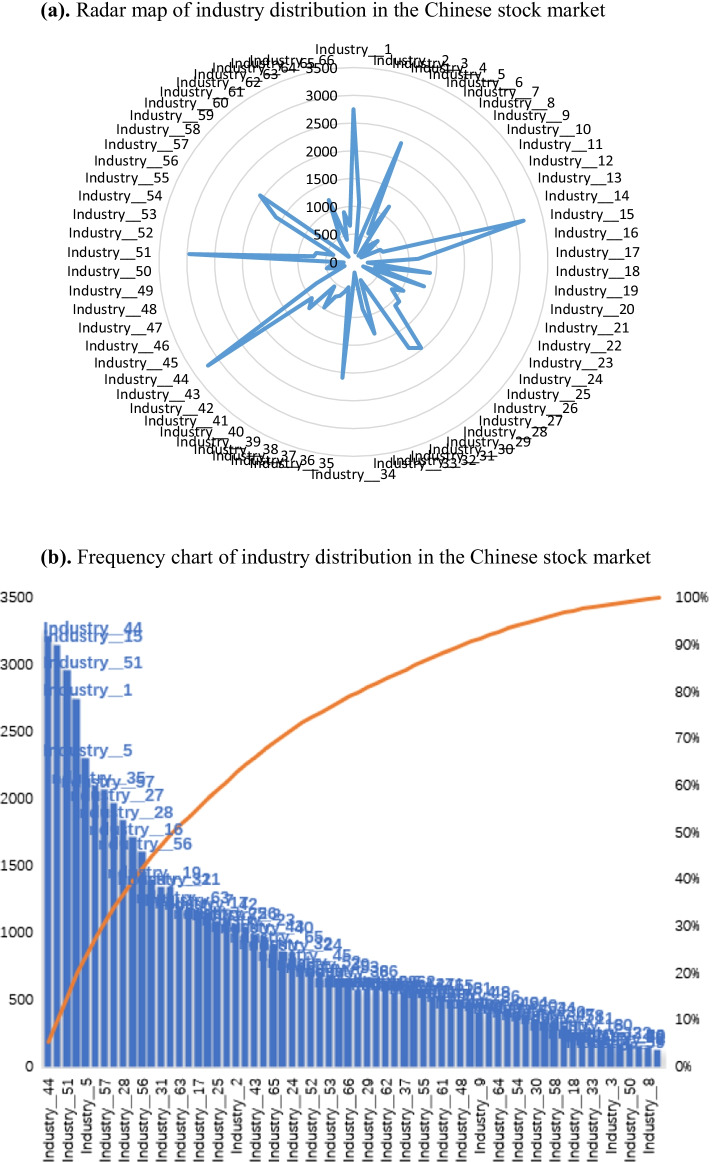
**a** Radar map of industry distribution in the Chinese stock market. **b** Frequency chart of industry distribution in the Chinese stock market. *Note*: See Table 1 for the specific names of the 66 industries

Industries are divided into 66 different sectors according to the CSRC, and the industry distributions are shown in Fig. 2a, b. The names of the 66 specific industries are listed in Table 1. In this study, we created dummy variables to examine whether the target industry was significantly affected by the pandemic. That is, ${Ind}_{N}$=1 if it is in the industry and 0 otherwise, where *N* = 1, 2,…,66.

**Table 1:** Specific names of industrial dummy variables

Industrial dummy	Specific name	Industrial dummy	Specific name	Industrial dummy	Specific name
Industry_1	Specialized equipment	Industry_23	National defense	Industry_45	White goods
Industry_2	Traditional Chinese medicine	Industry_24	Basic chemistry	Industry_46	Petroleum mining
Industry_3	Transportation equipment service	Industry_25	Household light industry	Industry_47	Planting and forestry
Industry_4	Instruments and apparatuses	Industry_26	Building materials	Industry_48	Textile manufacturing
Industry_5	Media	Industry_27	Building decoration	Industry_49	Comprehensive
Industry_6	Insurance and others	Industry_28	Real estate development	Industry_50	Audio-visual equipment
Industry_7	Optics and optoelectronics	Industry_29	New materials	Industry_51	Computer application
Industry_8	Public transport	Industry_30	Tourism	Industry_52	Computer equipment
Industry_9	Highway and railway transportation	Industry_31	Non-ferrous smelting and processing	Industry_53	Securities
Industry_10	Other electronics	Industry_32	Clothing and home textile	Industry_54	Trade
Industry_11	Breading industry	Industry_33	Airport shipping	Industry_55	Communication service
Industry_12	Agricultural service	Industry_34	Automobile	Industry_56	Communication equipment
Industry_13	Agricultural products process	Industry_35	Auto parts	Industry_57	General equipment
Industry_14	Packaging and printing	Industry_36	Port shipping	Industry_58	Papermaking
Industry_15	Chemical products	Industry_37	Coal mining and processing	Industry_59	Hotel and catering
Industry_16	Chemical pharmacy	Industry_38	Gas and water affairs	Industry_60	Mining service
Industry_17	Chemical synthetic materials	Industry_39	Logistics	Industry_61	Steel
Industry_18	New chemical materials	Industry_40	Environmental protection engineering	Industry_62	Bank
Industry_19	Medical equipment services	Industry_41	Biological products	Industry_63	Retail
Industry_20	Pharmaceutical business	Industry_42	Electric power	Industry_64	Non-automobile transportation
Industry_21	Semiconductor and its components	Industry_43	Electronic manufacturing	Industry_65	Food processing manufacturing
Industry_22	Park development	Industry_44	Electrical equipment	Industry_66	Beverage manufacturing

*Source*: China Securities Regulatory Commission

### The dependent and independent variables

The dependent variable in this study is the daily rate of return, which is calculated using the following simple method:

27 $$R_{t} = \frac{{P_{t} - P_{t - 1} }}{{P_{t - 1} }}.$$

where ${p}_{t}$ is the closing price of the stock on day *t,* and ${p}_{t-1}$ is the closing price of the stock on day *t-*1.

The explanatory variables were divided into four groups. The first group is the financial performance of the listed indicators, including the total revenue, operating cost, and operating profit. The second group is the performance of the stock market, in which the variables include the P/B ratio, buying volume, total market capitalization, change and percentage change in the price of a stock. The third group covered COVID-19 conditions, such as daily rates of infection, suspected infection, ICU patients, deaths, and recovery. The fourth group concerns how industries are distributed, as categorized by the CSRC.

Table 2 presents the descriptive statistics of the main variables used.

**Table 2:** Summary statistics

Variable	Explanation	Unit	Max	Min	Mean	Std
Daily_infection	Daily rates of infection of COVID-19 (i.e., confirmed cases)	Cases	77,262	17,238	51,105.063	21,523.618
Suspected_infection	Total number of suspected infection of COVID-19	Cases	26,359	3,434	15,511	8,368.905
ICU_patients	Total number of ICU patients of COVID-19	Cases	11,977	2,296	7,640.875	3,358.452
Death_cases	Total number of death cases of COVID-19	Cases	2,595	361	1,310.938	701.169
Recovery	Total number of recovery of COVID-19	Cases	24,758	475	7,906.188	7,181.045
Total_revenue	The overall measure of all sources of a company's income	RMB 10,000 yuan	97,360,000	− 36,100	854,738.899	4,046,225.602
Operating_cost	Costs incurred by the enterprise in all its business	RMB 10,000 yuan	91,950,000	769	752,321.270	3,472,541.850
Operating _profit	Profits obtained by the enterprise in all its business	RMB 10,000 yuan	31,210,000	− 385,100	116,538.292	985,136.004
Price_to_book_ratio	A stock's capitalization divided by its book value	%	278.600	0.321	4.049	8.592
Buy_vol	Number of stocks bought in marketplaces on a daily basis	100 shares	90,989,970	50	353,349.360	2,304,268.882
Total_market_capitalization	Total value of a company's securities as quoted on a stock market	RMB yuan	1.94E + 12	729,540,000	18,372,506,705	73,987,013,699
Change	The size of change in the price of a stock	RMB yuan per share	63.520	− 49.800	0.117	1.624
Pct_chg	The percentage change in the price of a stock	%	108.544	− 27.120	0.477	4.149
Close	The daily closing price of a stock	RMB yuan per share	1,118.000	1	17.071	32.231

## Results

The estimation process is not easy for this study. Since the sample size is relatively large, substantial time is needed to run each round of estimation. For example, it may take tens of minutes even for the simplest ordinary least squares (OLS) regression in a high-configuration desktop computer. After numerous rounds of tests, we present the following empirical results.

### Pure empirical results

To present the influence of COVID-19 on the Chinese stock market at a glance, we first show a group of estimation results without any guidance from the theoretical model. The purpose of this section is to determine whether our “intuition” works in reality.

In Table 3, we show a group of models using selected variables for stock fundamentals, as well as introduce the log forms for the pandemic variables. As shown in Table 3, the variables for the COVID-19 pandemic are very significant, although some of the signs are not consistent with our expectations. Interestingly, after adding the AR (1) term to the models, the signs of the pandemic variables are more consistent with our expectations. Logically speaking, we expect negative signs for daily rates of infection, suspected infection, ICU patients, and death. Indeed, the negative signs here are strong signals of a panicked attitude toward the COVID-19 pandemic. In contrast, the coefficient of recovery is positive, showing promises or optimistic attitudes regarding the pandemic.

**Table 3:** Empirical estimation results with dependent variable Pct_chg of pooled or panel least square with or without AR(1) term (*n* = 60,144)

	Model (3–1) (Pooled least squares)	Model (3–2) (Cross-section random effects)	Model (3–3) (Cross-section weights)	Model (3–4) (Period weights)	Model (3–5) (Period SUR)	Model (3–6) (Pooled least squares with AR(1))	Model (3–7) (Cross-section weights with AR(1))
ln(Daily_infection)	− 0.881*** (− 4.536)	− 0.881*** (− 4.490)	− 1.749*** (− 13.043)	− 3.073*** (− 17.921)	− 1.599*** (− 10.288)	− 3.559*** (− 18.671)	− 3.844*** (− 31.033)
ln(Suspected_infection)	0.941*** (18.893)	0.941*** (18.705)	1.039*** (30.261)	0.294*** (5.996)	0.714*** (15.472)	− 0.386*** (− 6.726)	− 0.118*** (− 3.177)
ln(ICU_patients)	− 3.282*** (− 19.063)	− 3.282*** (− 18.874)	− 1.445*** (− 12.163)	− 1.612*** (− 10.461)	− 2.283*** (− 15.709)	− 1.835*** (− 11.289)	− 0.390*** (− 3.637)
ln(Death_cases)	− 3.379*** (− 44.406)	− 3.379*** (− 43.964)	− 2.895*** (− 55.124)	− 2.682*** (− 39.619)	− 2.847*** (− 44.881)	− 2.098*** (− 28.328)	− 1.885*** (− 36.972)
ln(Recovery)	3.882*** (53.032)	3.882*** (52.504)	3.400*** (67.299)	3.248*** (47.551)	3.411*** (53.808)	2.656*** (36.069)	2.379*** (48.062)
Total_revenue	− 1.09E−08** (− 2.129)	− 1.09E−08** (− 2.108)	− 5.85E−09** (− 2.124)	− 1.08E−08** (− 2.281)	− 9.53E−09** (− 2.350)	− 1.14E−08** (− 2.179)	− 6.22E−09** (− 2.347)
Operating_cost	− 0.051*** (− 3.011)	− 0.051*** (− 2.981)	− 0.032*** (− 2.734)	− 0.049*** (− 3.182)	− 0.039*** (− 2.999)	− 0.039** (− 2.323)	− 0.035*** (− 3.387)
Operating_profit	− 3.45E−08* (− 1.649)	− 3.45E−08* (− 1.633)	− 1.79E−08* (− 1.819)	− 2.34E−08 (− 1.210)	− 3.16E−08* (− 1.909)	− 1.56E−08 (− 0.728)	− 6.57E−09 (− 0.702)
ln(Price_to_Book_Ratio)	0.212*** (8.256)	0.212*** (8.174)	0.134*** (6.989)	0.199*** (8.399)	0.198*** (9.737)	0.195*** (7.411)	0.121*** (7.061)
ln(Buy_vol)	0.046*** (6.480)	0.046*** (6.416)	0.019*** (3.479)	0.059*** (8.986)	0.039*** (6.977)	0.072*** (9.813)	0.039*** (7.940)
ln (Total_market_capitalization)	0.158*** (7.043)	0.158*** (6.973)	0.084*** (5.289)	0.077*** (3.735)	0.123*** (6.923)	0.015 (0.643)	− 0.006 (− 0.455)
Industry_2	− 0.761*** (− 6.444)	− 0.761*** (− 6.379)	− 0.561*** (− 5.913)	− 0.949*** (− 8.678)	− 0.696*** (− 7.443)	− 1.145*** (− 9.481)	− 0.885*** (− 10.626)
Industry_7			0.325*** (3.689)	0.416*** (3.890)	0.299*** (3.277)	0.419*** (3.547)	0.452*** (5.727)
Industry_9	− 0.679*** (− 3.891)	− 0.679*** (− 3.852)	− 0.438*** (− 5.168)	− 0.762*** (− 4.712)	− 0.626*** (− 4.522)	− 0.815*** (− 4.559)	− 0.602*** (− 7.364)
Industry_10	0.375** (2.465)	0.375** (2.440)	0.384*** (2.709)	0.375*** (2.661)	0.297** (2.462)	0.297* (1.906)	0.426*** (3.941)
Industry_16	− 0.472*** (− 4.648)	− 0.472*** (− 4.601)	− 0.336*** (− 3.771)	− 0.654*** (− 6.952)	− 0.476*** (− 5.909)	− 0.831*** (− 7.993)	− 0.714*** (− 9.303)
Industry_19	− 0.471*** (− 4.329)	− 0.471*** (− 4.287)	− 0.298*** (− 2.985)	− 0.657*** (− 6.517)	− 0.525*** (− 6.092)	− 0.793*** (− 7.114)	− 0.589*** (− 6.457)
Industry_20	− 0.863*** (− 4.874)	− 0.863*** (− 4.826)	− 0.669*** (− 4.612)	− 0.997*** (− 6.077)	− 0.868*** (− 6.185)	− 1.156*** (− 6.382)	− 0.864*** (− 6.526)
Industry_21	0.791*** (7.055)	0.791*** (6.984)	1.048*** (10.079)	0.976*** (9.391)	0.755*** (8.501)	0.933*** (8.131)	1.108*** (11.187)
Industry_22	− 0.619** (− 2.528)	− 0.619** (− 2.503)	− 0.413*** (− 2.809)	− 0.513** (− 2.259)	− 0.482** (− 2.479)	− 0.434* (− 1.728)	− 0.269** (− 2.103)
Industry_25	− 0.305** (− 2.569)	− 0.305** (− 2.543)	− 0.194** (− 2.479)	− 0.274** (− 2.492)	− 0.225** (− 2.387)	− 0.258** (− 2.122)	− 0.197** (− 2.793)
Industry_27	− 0.334*** (− 3.406)	− 0.334*** (− 3.372)	− 0.212*** (− 3.581)	− 0.309*** (− 3.400)	− 0.277*** (− 3.562)	− 0.266*** (− 2.652)	− 0.203*** (− 3.839)
Industry_28	− 0.436*** (− 4.345)	− 0.436*** (− 4.302)	− 0.288*** (− 4.605)	− 0.398*** (− 4.286)	− 0.310*** (− 3.902)	− 0.403*** (− 3.924)	− 0.316*** (− 5.528)
Industry_29	0.289* (1.923)	0.289* (1.904)	0.437*** (3.356)	0.419*** (3.011)	0.265** (2.231)	0.330** (2.149)	0.441*** (3.891)
Industry_32	− 0.385*** (− 3.012)	− 0.385*** (− 2.982)	− 0.274*** (− 3.286)	− 0.421*** (− 3.556)	− 0.411*** (− 4.062)	− 0.398*** (− 3.042)	− 0.339*** (− 4.579)
Industry_33	− 0.600** (− 2.444)	− 0.600** (− 2.419)	− 0.325* (− 1.832)	− 0.581** (− 2.552)	− 0.349* (− 1.795)	− 0.532** (− 2.114)	− 0.311* (− 1.823)
Industry_36	− 0.838*** (− 5.051)	− 0.838*** (− 5.000)	− 0.544*** (− 5.867)	− 0.703*** (− 4.573)	− 0.635*** (− 4.832)	− 0.649*** (− 3.827)	− 0.447*** (− 5.341)
Industry_37	− 0.367** (− 2.384)	− 0.367** (− 2.360)	− 0.261*** (− 3.324)	− 0.388*** (− 2.715)	− 0.243** (− 1.989)	− 0.395** (− 2.503)	− 0.353*** (− 4.611)
Industry_38	− 0.423** (− 2.969)	− 0.423*** (− 2.940)	− 0.326** (− 4.071)	− 0.468*** (− 3.542)	− 0.405*** (− 3.585)	− 0.446*** (− 3.053)	− 0.385*** (− 5.547)
Industry_39	− 0.284*** (− 1.998)	− 0.284** (− 1.978)	− 0.197* (− 1.924)	− 0.265** (− 2.013)			
Industry_41	− 0.670** (− 4.331)	− 0.670*** (− 4.288)	− 0.296* (− 1.959)	− 0.799*** (− 5.574)	− 0.748*** (− 6.104)	− 0.957*** (− 6.034)	− 0.644*** (− 4.785)
Industry_42	− 0.512*** (− 4.394)	− 0.512*** (− 4.350)	− 0.321*** (− 5.095)	− 0.531*** (− 4.919)	− 0.445*** (− 4.819)	− 0.559*** (− 4.687)	− 0.437*** (− 7.288)
Industry_43	0.254** (2.053)	0.254** (2.033)	0.489*** (5.095)	0.319*** (2.788)	0.338*** (3.450)	0.360*** (2.844)	0.522*** (6.279)
Industry_46	− 0.566** (− 2.213)	− 0.566** (− 2.191)	− 0.431*** (− 2.704)	− 0.606** (− 2.558)	− 0.488** (− 2.409)	− 0.606** (− 2.315)	− 0.455*** (− 3.207)
Industry_49	− 0.362** (− 2.032)	− 0.362** (− 2.012)	− 0.305** (− 2.566)	− 0.472*** (− 2.859)	− 0.356** (− 2.522)	− 0.385** (− 2.315)	− 0.240** (− 2.313)
Industry_50	0.618** (2.424)	0.618** (2.399)	0.404* (1.914)	0.651*** (2.756)	0.620*** (3.071)	0.621** (2.381)	0.524*** (2.933)
Industry_51	0.575*** (6.464)	0.575*** (6.399)	0.687*** (9.381)	0.633*** (7.676)	0.448*** (6.356)	0.555*** (6.097)	0.655*** (10.012)
Industry_52	0.498*** (3.619)	0.498*** (3.583)	0.595*** (4.881)	0.687*** (5.391)	0.467*** (4.288)	0.684*** (4.863)	0.679*** (6.771)
Industry_53	0.453*** (2.908)						0.235** (2.115)
Industry_54	− 0.462** (− 2.508)	− 0.462** (− 2.483)	− 0.291** (− 2.529)	− 0.449*** (− 2.627)	− 0.441*** (− 3.019)	− 0.434** (− 2.302)	− 0.307*** (− 2.957)
Industry_55	0.495*** (3.171)	0.495*** (3.139)	0.529*** (4.026)	0.452*** (3.126)	0.431*** (3.481)	0.476*** (2.981)	0.490*** (3.830)
Industry_56	0.473*** (4.527)	0.473*** (4.482)	0.594*** (6.744)	0.521*** (5.383)	0.442*** (5.346)	0.607*** (5.681)	0.728*** (9.998)
Industry_59	− 0.810*** (− 2.739)	− 0.810*** (− 2.711)	− 0.476* (− 1.852)	− 0.764*** (− 2.788)	− 0.594** (− 2.534)	− 0.642** (− 2.119)	
Industry_60	− 0.693*** (− 3.242)	− 0.693*** (− 3.209)	− 0.477*** (− 3.324)	− 0.589** (− 2.978)	− 0.613*** (− 3.622)	− 0.613*** (− 2.801)	− 0.413*** (− 3.214)
Industry_61	− 0.401** (− 2.528)	− 0.401** (− 2.502)	− 0.266*** (− 3.110)	− 0.508*** (− 3.452)	− 0.216*** (− 1.716)	− 0.476*** (− 2.930)	− 0.350*** (− 4.214)
Industry_62	− 0.651*** (− 3.784)	− 0.651*** (− 3.746)	− 0.326*** (− 4.898)	− 0.680*** (− 4.267)	− 0.492*** (− 3.606)	− 0.693*** (− 3.932)	− 0.542*** (− 6.326)
Industry_63	− 0.390*** (− 3.399)	− 0.390*** (− 3.746)	− 0.272*** (− 3.932)	− 0.424*** (− 3.986)	− 0.376*** (− 4.133)	− 0.384*** (− 3.268)	− 0.343*** (− 5.374)
Industry_66	− 0.762*** (− 5.339)	− 0.762*** (− 5.286)	− 0.451*** (− 5.425)	− 0.728*** (− 5.505)	− 0.594*** (− 5.249)	− 0.609*** (− 4.169)	− 0.438*** (− 5.944)
AR(1)						0.068*** (16.825)	− 0.002 (− 0.566)
Random effect (Cross)		Yes					
Adjusted *R*^2^	0.076	0.076	0.108	0.095	0.086	0.103	0.143

The values of the constant terms are not reported. *t* statistics in parentheses. For industrial variables, only those who are statistically significant are reported in the table

***p ≤ 0.01, ** 0.01 < *p* < 0.05, *0.05 < *p* < 0.1

Fortunately, many of the industry dummies are significant, which is a promising result, even in our casual attempt. As shown in the last column of Table 3, for the model with both the AR (1) term and cross-section weights, all five pandemic variables are statistically significant, and the signs are exactly as expected. Although the AR (1) term is not significant here, it brings us the largest adjusted *R*^2^ value in this group of models, which has been our best fit so far.

### Estimation with partial theoretical guidance

The guidance of the theoretical model shown earlier makes us more confident about the variables to be included. However, many variables have a value that is either negative or zero, so we cannot take the log form of all the variables, as suggested by the theory. For example, the change in the number of suspected infections, as well as that of ICU patients, has some zero values. Therefore, we have to seek a compromise in which we do not take the log form for some key variables. Hence, we consider this group of model estimations with “partial” theoretical guidance.

As shown in Table 4, the overall fitness of this group of models is much better, as the adjusted *R*^*2*^ values tend to be much larger. Notably, models (4–7) presented in the last column of Table 4 show an adjusted *R*^*2*^ value of 0.333, which is considered extraordinarily high in stock market studies. However, in this group of models, the signs of the estimation coefficients are not easy to understand. According to Eq. (24) in the theoretical section, for all their original forms, we would expect a negative sign for daily rates of infection, suspected infection, ICU patients, as well as deaths, and a positive sign for recovery. In addition, we would expect a positive sign for the log of the difference in the pandemic variable(s), regardless of the specific variables we use here. As shown in Table 4, the one with only AR (1) appears to have a better sign in the log of the difference in the daily rates of infection. However, the signs of death and recovery are not consistent with our expectations. Moreover, although the theory suggests that the log of the stock price (i.e., “close” as the variable name) be negative, we end up with a positive coefficient in all the models in this group.

**Table 4:** Empirical estimation results with dependent variable Pct_chg of pooled or panel least square with or without AR(1) term with partial theoretical guidance (*n* = 60,144)

	Model (4–1) (Pooled Least Squares)	Model (4–2) (Cross-section random effects)	Model (4–3) (Cross-section weights)	Model (4–4) (Period weights)	Model (4− 5) (Period SUR)	Model (4–6) (Pooled least squares with AR(1))	Model (4–7) (Cross-section weights with AR(1))
ln(Close)	0.409*** (21.608)	0.409*** (22.533)	0.264*** (17.958)	0.296*** (17.059)	0.332*** (21.147)	0.257*** (13.575)	0.409*** (21.608)
Daily_infection	7.56E−05*** (3.980)	7.56E−05*** (4.150)	0.000*** (14.494)	− 0.000*** (− 14.692)	− 8.76E−05*** (− 4.825)	− 0.000*** (− 40.643)	7.56E−05*** (3.980)
ln(Daily_infection)	− 1.067*** (− 18.045)	− 1.067*** (− 18.817)	− 1.066*** (− 41.924)	− 0.615*** (− 11.808)	− 0.905*** (− 17.276)	0.271*** (4.832)	− 1.067*** (− 18.045)
Suspected_infection	− 5.67E−05*** (− 4.704)	− 5.67E−05*** (− 4.906)	− 6.56E−05*** (− 8.408)	− 0.000*** (− 17.494)	− 0.000*** (− 11.284)	− 0.001*** (− 48.273)	− 5.67E−05*** (− 4.704)
∆Suspected_infection	5.51E−05** (2.065)	5.51E−05** (2.153)	− 7.81E−05** (− 4.521)	0.000*** (14.279)	0.000*** (7.582)	0.001*** (41.419)	5.51E−05** (2.065)
ICU_patients	− 0.001*** (− 33.086)	− 0.001*** (− 34.502)	− 0.001*** (− 35.557)	− 0.001*** (− 39.914)	− 0.001*** (− 32.301)	− 0.002*** (− 57.220)	− 0.001*** (− 33.086)
∆ICU_patients	0.001*** (22.335)	0.001*** (23.291)	0.001*** (20.854)	0.002*** (34.307)	0.001*** (25.006)	0.001*** (22.335)	0.001*** (22.335)
Death_cases	− 0.000 (− 0.106)	− 0.000 (− 0.111)	− 0.009*** (− 10.677)	0.021*** (16.966)	0.009*** (7.597)	− 0.000 (− 0.106)	− 0.000 (− 0.106)
ln(∆Death_cases)	− 2.349*** (− 20.847)	− 2.349*** (− 21.739)	− 1.501*** (− 20.568)	− 1.226*** (− 11.346)	− 1.586*** (− 15.268)	− 2.349*** (− 20.847)	− 2.349*** (− 20.847)
Recovery	− 4.61E−05 (− 0.783)	− 4.61E−05 (− 0.816)	0.000*** (5.475)	− 0.001*** (− 17.720)	− 0.000*** (− 9.512)	− 4.61E−05 (− 0.783)	− 4.61E−05 (− 0.783)
ln(∆Recovery)	3.086*** (35.279)	3.086*** (36.789)	3.123*** (55.130)	1.990*** (23.765)	2.487*** (31.882)	3.086*** (35.279)	3.086*** (35.279)
Total_revenue	− 1.09E−08** (− 2.170)	− 1.09E−08** (− 2.263)	− 5.47E−09** (− 2.163)	− 1.09E−08** (− 2.367)	− 9.36E−09** (− 2.259)	− 1.09E−08** (− 2.170)	− 1.09E−08** (− 2.170)
ln(Operating_cost)	− 0.028** (− 2.555)	− 0.028*** (− 2.665)	− 0.026*** (− 3.651)	− 0.058*** (− 5.881)	− 0.027*** (− 3.014)	− 0.028** (− 2.555)	− 0.028** (− 2.555)
Operating_profit	− 2.09E−08 (− 1.035)	− 2.09E−08 (− 1.079)	− 1.58E−08 (− 1.734)	− 2.22E−08 (− 1.200)	− 2.16E−08 (− 1.294)	− 2.09E−08 (− 1.035)	− 2.09E−08 (− 1.035)
ln(Buy_vol)	0.117*** (14.523)	0.117*** (15.145)	0.067*** (11.331)	0.112*** (15.116)	0.097*** (14.458)	0.117*** (14.523)	0.117*** (14.523)
Industry_2	− 0.781*** (− 6.769)	− 0.781*** (− 7.058)	− 0.582*** (− 6.389)	− 0.938*** (− 8.857)	− 0.702*** (− 7.348)	− 1.168*** (− 10.135)	− 0.881*** (− 12.056)
Industry_7	0.309*** (2.764)	0.309*** (2.882)	0.308*** (3.766)	0.483*** (4.703)	0.342*** (3.693)	0.436*** (3.892)	0.462*** (6.415)
Industry_9	− 0.521*** (− 3.086)	− 0.521*** (− 3.219)	− 0.329*** (− 4.384)	− 0.738*** (− 4.759)	− 0.543*** (− 3.881)	− 0.807*** (− 4.782)	− 0.550*** (− 7.754)
Industry_10	0.339** (2.312)	0.339** (2.411)	0.310** (2.427)	0.312** (2.315)	0.308** (2.537)	0.261* (1.778)	0.429*** (4.494)
Industry_12				− 0.552** (− 2.311)	− 0.409* (− 1.902)	− 0.548** (− 2.109)	− 0.397** (− 2.093)
Industry_16	− 0.484*** (− 4.896)	− 0.484*** (− 5.105)	− 0.352*** (− 4.106)	− 0.678*** (− 7.479)	− 0.455*** (− 5.562)	− 0.853*** (− 8.633)	− 0.716*** (− 10.474)
Industry_19	− 0.501*** (− 4.721)	− 0.501*** (− 4.923)	− 0.286*** (− 2.997)	− 0.635*** (− 6.524)	− 0.486*** (− 5.535)	− 0.839*** (− 7.922)	− 0.579*** (− 7.429)
Industry_20	− 1.017*** (− 5.886)	− 1.017*** (− 6.138)	− 0.778*** (− 5.705)	− 1.062*** (− 6.703)	− 0.970*** (− 6.785)	− 1.205*** (− 6.982)	− 0.824*** (− 7.554)
Industry_21	0.738*** (6.808)	0.738*** (7.099)	1.003*** (9.825)	0.903*** (9.073)	0.755*** (8.406)	0.869*** (8.026)	1.101*** (11.566)
Industry_22	− 0.544** (− 2.281)	− 0.544** (− 2.3678)	− 0.375*** (− 2.929)	− 0.375*** (− 2.939)	− 0.456** (− 2.307)	− 0.478** (− 2.003)	− 0.289*** (− 3.049)
Industry_23				0.395*** (3.688)	0.164* (1.691)	0.255** (2.189)	0.167** (2.351)
Industry_25	− 0.335*** (− 2.920)	− 0.335*** (− 3.045)	− 0.217*** (− 3.082)	− 0.276** (− 2.622)	− 0.266** (− 2.803)	− 0.279** (− 2.381)	− 0.188*** (− 3.258)
Industry_27	− 0.294*** (− 3.076)	− 0.294*** (− 3.208)	− 0.202*** (− 3.759)	− 0.279*** (− 3.175)	− 0.268*** (− 3.379)	− 0.218** (− 2.283)	− 0.169*** (− 3.803)
Industry_28	− 0.313*** (− 3.211)	− 0.313*** (− 3.349)	− 0.218*** (− 3.773)	− 0.364*** (− 4.070)	− 0.257*** (− 3.185)	− 0.362*** (− 3.708)	− 0.271*** (− 5.432)
Industry_29	0.338** (2.308)	0.339** (2.407)	0.460*** (3.624)	0.433*** (3.220)	0.318** (2.618)	0.344** (2.348)	0.491*** (4.934)
Industry_32	− 0.358*** (− 2.868)	− 0.358*** (− 2.991)	− 0.269*** (− 3.625)	− 0.399*** (− 3.481)	− 0.394*** (− 3.811)	− 0.372*** (− 2.983)	− 0.293*** (− 4.751)
Industry_33	− 0.551** (− 2.296)	− 0.551** (− 2.394)	− 0.327** (− 1.999)	− 0.591*** (− 2.685)	− 0.361* (− 1.818)	− 0.568** (− 2.369)	− 0.287** (− 1.958)
Industry_36	− 0.615*** (− 3.800)	− 0.615*** (− 3.963)	− 0.419*** (− 5.131)	− 0.553*** (− 3.724)	− 0.504*** (− 3.764)	− 0.568*** (− 3.510)	− 0.399*** (− 5.762)
Industry_37	− 0.290* (− 1.929)	− 0.290** (− 2.012)	− 0.234*** (− 3.337)	− 0.356*** (− 2.578)	− 0.221* (− 1.771)	− 0.360** (− 2.393)	− 0.336*** (− 5.274)
Industry_38	− 0.353** (− 2.535)	− 0.353** (− 2.644)	− 0.279*** (− 3.948)	− 0.429*** (− 3.356)	− 0.374*** (− 3.239)	− 0.418** (− 3.003)	− 0.344*** (− 6.209)
Industry_41	− 0.652*** (− 4.381)	− 0.652*** (− 4.569)	− 0.341** (− 2.370)	− 0.836*** (− 6.119)	− 0.691*** (− 5.609)	− 0.987*** (− 6.629)	− 0.647*** (− 5.427)
Industry_42	− 0.351*** (− 3.086)	− 0.351*** (− 3.218)	− 0.231*** (− 4.012)	− 0.441*** (− 4.228)	− 0.337*** (− 3.576)	− 0.489*** (− 4.311)	− 0.377*** (− 7.458)
Industry_43	0.207* (1.734)	0.207* (1.808)	0.432*** (4.660)	0.374*** (3.410)	0.325*** (3.280)	0.322*** (2.695)	0.523*** (6.985)
Industry_46	− 0.489* (− 1.958)	− 0.489** (− 2.042)	− 0.390** (− 2.736)	− 0.597*** (− 2.609)	− 0.465** (− 2.249)	− 0.617** (2.474)	− 0.435*** (− 3.432)
Industry_50	0.722*** (2.897)	0.722*** (3.021)	0.462*** (2.302)	0.741** (3.238)	0.707*** (3.424)	0.722*** (2.899)	0.620*** (3.901)
Industry_51	0.546*** (6.364)	0.546*** (6.637)	0.636*** (9.240)	0.599*** (7.601)	0.467*** (6.573)	0.504*** (5.880)	0.616*** (10.844)
Industry_52	0.525*** (3.910)	0.525*** (4.078)	0.589*** (4.988)	0.682*** (5.544)	0.514*** (4.630)	0.676*** (5.040)	0.667*** (7.340)
Industry_54	− 0.328* (− 1.868)	− 0.328* (− 1.948)	− 0.217** (− 2.179)	− 0.285* (− 1.772)	− 0.325** (− 2.236)		− 0.169** (− 2.089)
Industry_55	0.491*** (3.265)	0.491*** (3.405)	0.583*** (5.018)	0.496*** (3.589)	0.447*** (3.586)	0.436*** (2.899)	0.480*** (4.361)
Industry_56	0.421*** (4.169)	0.421*** (4.347)	0.563*** (6.618)	0.519*** (5.616)	0.431*** (5.158)	0.555*** (5.507)	0.719*** (10.672)
Industry_59	− 0.568** (− 1.962)	− 0.568** (− 2.046)		− 0.572** (− 2.154)	− 0.419* (− 1.752)		
Industry_60	− 0.469** (− 2.239)	− 0.469** (− 2.336)	− 0.321** (− 2.407)	− 0.438** (− 2.284)	− 0.434** (− 2.505)	− 0.463** (− 2.215)	− 0.316*** (− 3.143)
Indusytu_61	− 0.281* (− 1.180)	− 0.281* (− 1.888)	− 0.193* (− 2.502)	− 0.422*** (− 2.959)		− 0.395** (− 2.539)	− 0.297*** (− 3.860)
Industry_62	− 0.525*** (− 3.179)	− 0.525*** (− 3.315)	− 0.359*** (− 4.746)	− 0.611*** (− 4.028	− 0.425*** (− 3.110)	− 0.736*** (− 4.458)	− 0.555*** (− 8.023)
Industry_63	− 0.332*** (− 2.971)	− 0.332*** (− 3.098)	− 0.255*** (− 4.087)	− 0.359*** (− 3.506)	− 0.347*** (− 3.747)	− 0.332*** (− 2.977)	− 0.297*** (− 5.592)
Industry_66	− 0.754*** (− 5.414)	− 0.754*** (− 5.645)	− 0.443*** (− 5.864)	− 0.734*** (− 5.739)	− 0.614*** (− 5.322)	− 0.641*** (− 4.605)	− 0.423*** (− 6.798)
AR(1)						0.072*** (18.198)	− 0.005 (− 1.413)
Random effect (Cross)		Yes					
Adjusted *R*^2^	0.113	0.113	0.198	0.154	0.129	0.186	0.333

The values of the constant terms are not reported. *t* statistics in parentheses. For industrial variables, only those who are statistically significant are reported in the table

***p ≤ 0.01, ** 0.01 < *p* < 0.05, *0.05 < *p* < 0.1

### Estimation with complete theoretical guidance

To support and examine the theoretical model at its full strength, we used a bit of sleight of the hand. First, we introduce the quadratic form to the variables with negative values, the transformation of which is shown in Eq. (26), in the theoretical section. Second, we “manipulate” the data slightly by changing all the zero values of the key variables to a tiny value of 0.001. This manipulation does not change the essence of the data set but enables us to take the log forms. The results for this group of models are presented in Table 5.

**Table 5:** Empirical estimation results with dependent variable ln((Pct_chg)^2^) of pooled or panel least square with or without AR(1) term with complete theoretical guidance (*n* = 60,144)

	Model (5–1) (Pooled least squares)	Model (5–2) (Cross-section random effects)	Model (5–3) (Cross-section weights)	Model (5–4) (Period weights)	Model (5–5) (Period SUR)	Model (5–6) (Pooled least squares with AR(1))	Model (5–7) (Cross-section weights with AR(1))
ln((Close)^2^)	0.235*** (25.978)	0.254*** (21.598)	0.164*** (24.739)	0.177*** (20.876)	0.168*** (16.306)	0.246*** (24.736)	0.169*** (23.246)
Daily_infection	− 1.49E−05 (− 0.945)	− 1.51E−05 (− 0.996)	− 2.34E−05** (− 2.157)	− 3.43E−05** (− 2.150)	− 4.88E−05 (− 3.279)	5.44E−05*** (2.777)	5.82E−05*** (4.378)
ln(∆Daily_infection^2^)	0.091*** (2.582)	0.091*** (2.685)	0.105*** (4.337)	0.102*** (2.826)	0.104*** (2.927)	0.062* (1.752)	0.064*** (2.668)
Suspected_infection	− 0.000*** (− 33.811)	− 0.000*** (− 35.152)	− 0.000*** (− 46.005)	− 0.000*** (− 43.731)	− 0.000*** (− 43.146)	− 0.000*** (− 19.818)	− 0.000*** (− 26.181)
ln((∆Suspected_infection_new)^2^)	0.023*** (2.959)	0.023*** (3.079)	0.024*** (4.495)	0.039*** (5.168)	0.036*** (5.049)	− 0.003 (− 0.388)	− 0.008 (− 1.313)
ICU_patients	0.000*** (12.227)	0.000*** (12.696)	0.000*** (17.467)	0.000*** (10.443)	0.000*** (11.104)	0.000*** (10.779)	0.000*** (15.095)
ln((∆ICU_patients_new)^2^)	− 0.055*** (− 6.115)	− 0.055*** (− 6.355)	− 0.053*** (− 8.563)	− 0.072*** (− 7.891)	− 0.066*** (− 7.608)	− 0.029*** (− 3.049)	− 0.023*** (− 3.494)
Death_cases	− 0.009 (− 11.660)	− 0.009 (− 12.102)	− 0.008*** (− 15.112)	− 0.009*** (− 10.205)	− 0.008*** (− 9.604)	− 0.012*** (− 13.024)	− 0.011*** (− 18.265)
ln((∆Death_cases)^2^)	0.772*** (12.361)	0.773*** (12.845)	0.632*** (14.677)	0.734*** (11.831)	0.712*** (11.287)	0.779*** (12.457)	0.661*** (15.612)
Recovery	0.000*** (9.244)	0.000*** (9.589)	0.000*** (12.040)	0.000*** (7.429)	0.000*** (6.812)	0.000*** (10.804)	0.000*** (15.612)
ln((∆Recovery)^2^)	− 0.050 (− 1.241)	− 0.051 (− 1.298)	− 0.016 (− 0.566)	0.016 (0.429)	0.014 (0.405)	− 0.019 (− 0.454)	0.014 (0.516)
Total_revenue	− 9.97E−09*** (− 2.082)	− 1.03E−08*** (− 1.649)	− 8.55E−09** (− 2.153)	− 1.01E−08** (− 2.261)	− 9.42E−09* (− 1.725)	− 6.21E−09 (− 1.178)	− 5.29E−09 (− 1.507)
ln(Operating_cost)	− 0.107*** (− 10.388)	− 0.109*** (− 8.129)	− 0.101*** (− 14.053)	− 0.111*** (− 11.538)	− 0.107*** (− 9.121)	− 0.101*** (− 8.929)	− 0.094*** (− 12.039)
Operating_profit	− 7.28E−08*** (− 3.767)	− 7.42E−08*** (− 2.957)	− 8.74E−08*** (− 4.204)	− 7.02E−08*** (− 3.889)	− 6.96E−08*** (− 3.163)	− 8.94E−08*** (− 4.206)	− 1.09E−07** (− 4.967)
ln(Buy_vol)	0.147** (19.098)	0.156*** (15.568)	0.551*** (9.512)	0.124*** (17.105)	0.123*** (13.926)	0.668*** (7.007)	0.152*** (24.806)
Industry_5	0.655*** (7.554)	0.656*** (5.823)	− 0.176* (− 1.876)	0.575*** (7.091)	0.507*** (5.136)	0.465*** (3.946)	0.574*** (9.018)
Industry_7	0.416*** (3.879)	0.408*** (2.925)	0.369*** (5.031)	0.345*** (3.439)	0.364*** (2.977)	− 0.932*** (− 3.234)	− 0.191** (− 1.918)
Industry_8	− 0.794*** (− 3.028)	− 0.767** (− 2.253)		− 0.548** (− 2.235)		− 0.932*** (− 3.234)	0.392*** (4.894)
Industry_9	− 1.513*** (− 9.354)	− 1.489*** (− 7.089)	− 1.301*** (− 8.246)	− 1.597*** (− 10.559)	− 1.601*** (− 8.691)	− 1.444*** (− 8.119)	− 1.208*** (− 7.289)
Industry_10	0.590*** (4.200)	0.574*** (3.144)	0.505*** (5.143)	0.496*** (3.771)	0.474*** (2.961)	0.613*** (3.964)	0.532*** (4.936)
Industry_15	0.177** (2.202)	0.175* (1.683)		0.158** (2.100)		0.209** (2.379)	0.105* (1.688)
Industry_16	0.225** (2.375)	0.211* (1.718)	0.141** (2.059)			0.247** (2.376)	0.176** (2.449)
Industry_19	0.238*** (2.343)		0.241*** (3.299)	0.196** (2.068)		0.248** (2.221)	0.264*** (3.257)
Industry_21	0.524*** (5.047)	0.484*** (3.591)	0.525*** (7.094)	0.379*** (3.905)	0.363*** (3.068)	0.571*** (5.001)	0.586*** (7.132)
Industry_22	− 0.627*** (− 2.744)	− 0.625** (− 2.105)	− 1.029*** (− 6.274)	− 0.502** (− 2.351)	0.379*** (2.976)	− 0.694*** (− 2.761)	− 1.068*** (− 5.909)
Industry_23	0.267** (2.385)	0.252* (1.734)	0.131* (1.681)	0.343*** (3.276)	− 0.319** (− 2.546)	0.279** (2.269)	0.148* (1.723)
Industry_26	− 0.205* (− 1.867)		− 0.365*** (− 4.907)	− 0.300*** (− 2.919)	− 0.319** (− 2.546)		− 0.368*** (− 4.498)
Industry_27	− 0.589*** (− 6.437)	− 0.575*** (− 4.831)	− 0.583* (− 8.891)	− 0.562*** (− 6.559)	− 0.514*** (− 4.922)	− 0.590*** (− 5.864)	− 0.574*** (− 8.145)
Industry_28	− 0.714*** (− 7.644)	− 0.694*** (− 5.723)	− 0.634*** (− 8.891)	− 0.824*** (− 9.438)	− 0.778*** (− 7.313)	− 0.718*** (− 6.993)	− 0.621*** (− 7.677)
Industry_29	0.579*** (4.122)	0.569*** (3.120)	0.442*** (4.761)	0.505*** (3.847)	0.459*** (2.869)	0.618*** (4.004)	0.492*** (4.792)
Industry_31			− 0.201*** (− 2.663)	− 0.202** (− 2.092)	− 0.289** (− 2.037)		− 0.153* (− 1.841)
Industry_34	0.330* (1.953)		0.199** (1.701)	0.277* (1.753)	0.346* (1.798)		0.244* (1.960)
Industry_36	− 1.037*** (− 6.693)	− 1.015*** (− 5.042)	− 0.759*** (− 6.339)	− 0.802*** (− 5.541)	− 0.551*** (− 3.123)	− 1.124*** (− 6.602)	− 0.819*** (− 6.253)
Industry_37	− 0.757*** (− 5.256)	− 0.736*** (− 3.933)	− 0.849*** (− 8.504)	− 0.763*** (− 5.661)	− 0.722*** (− 4.401)	− 0.679*** (− 4.285)	− 0.769*** (− 7.432)
Industry_38	− 0.785*** (− 5.887)	− 0.771*** (− 4.451)	− 0.894*** (− 9.921)	− 0.721*** (− 5.781)	− 0.661*** (− 4.353)	− 0.813*** (− 5.545)	− 0.902*** (− 9.297)
Industry_42	− 1.004*** (− 9.224)	− 0.983*** (− 6.952)	− 0.809*** (− 9.501)	− 0.959*** (− 9.429)	− 0.876*** (− 7.066)	− 0.957*** (− 7.995)	0.350*** (3.414)
Industry_43	0.328*** (2.859)	0.299** (2.013)	0.274*** (3.543)	0.333*** (3.107)	0.391*** (2.998)	0.348*** (2.766)	0.306*** (3.628)
Industry_46	0.453*** (2.908)	− 0.928*** (− 2.988)	− 0.565*** (− 2.899)	− 0.894*** (− 4.001)	− 0.820*** (− 3.013)	− 0.882*** (− 3.356)	− 0.554*** (− 2.651)
Industry_47	0.555*** (3.133)	0.557** (2.419)	0.486*** (4.178)	0.534*** (3.224)	0.556*** (2.755)	0.625** (3.208)	0.559*** (4.365)
Industry_50	0.565** (2.365)	0.572* (1.844)	0.503*** (2.817)	0.593*** (2.658)	0.603** (2.216)	0.271* (1.647)	0.535*** (2.721)
Industry_51	0.691*** (8.408)	0.666*** (6.239)	0.667*** (11.864)	0.592*** (7.709)	0.564*** (6.027)	0.741*** (8.202)	0.732*** (11.871)
Industry_52	0.842*** (6.559)	0.827*** (4.957)	0.608*** (7.442)	0.592*** (7.709)	0.675*** (4.612)	0.889*** (6.299)	0.656*** (7.231)
Industry_53	− 0.523*** (− 3.899)	− 0.536** (− 3.077)	− 0.509*** (− 4.790)	− 0.348*** (− 2.773)	− 0.279* (− 1.827)	− 0.456*** (− 3.093)	− 0.443*** (− 3.754)
Industry_54	− 0.499*** (− 2.976)	− 0.476** (− 2.184)	− 0.384*** (− 2.764)	− 0.485*** (− 3.391)	− 0.416** (− 2.175)	− 0.524*** (− 2.839)	− 0.378** (− 2.484)
Industry_55	0.591*** (4.102)	0.574*** (3.065)	0.619*** (6.328)	0.519*** (3.859)	0.483*** (2.947)	0.668*** (4.219)	0.702*** (6.531)
Industry_56	0.600*** (6.219)	0.584*** (4.660)	0.476*** (7.112)	0.551*** (6.103)	0.593*** (5.394)	0.609*** (5.742)	0.488*** (6.581)
Industry_58	− 0.576*** (− 3.174)	− 0.561** (− 3.067)	− 0.541*** (− 3.630)		− 0.376* (− 1.819)	− 0.558*** (− 2.799)	− 0.539*** (− 3.269)
Industry_60	− 0.418** (− 2.089)		− 0.440*** (− 2.861)	− 0.355* (− 1.895)		− 0.480** (− 2.181)	− 0.506*** (− 2.969)
Industry_61	− 0.615*** (− 4.132)	− 0.593*** (− 3.067)	− 0.620*** (− 5.955)	− 0.637*** (− 4.581)	− 0.563*** (− 3.323)	− 0.591*** (− 3.610)	− 0.608*** (− 5.421)
Industry_62	− 1.005*** (− 6.354)	− 0.993*** (− 4.831)	− 1.157*** (− 11.701)	− 0.923*** (− 6.246)	− 0.817*** (− 4.534)	− 0.359*** (− 3.052)	− 1.162*** (− 10.533)
Industry_63	− 0.353*** (− 3.298)	− 0.336** (− 2.419)	− 0.371*** (− 4.764)	− 0.298*** (− 2.983)	− 0.239* (− 1.958)	− 0.359*** (− 3.052)	− 0.359*** (− 4.258)
Industry_66	− 1.123*** (− 8.421)	− 1.134*** (− 6.540)	− 0.982*** (− 9.668)	− 1.065*** (− 8.541)	− 0.993*** (− 6.534)	− 1.119*** (− 7.636)	
AR(1)						0.047*** (10.629)	0.058*** (13.317)
Random effect (cross)		Yes					
Adjusted *R*^2^	0.116	0.099	0.173	0.128	0.101	0.103	0.156

The values of the constant terms are not reported. *t* statistics in parentheses. For industrial variables, only those who are statistically significant are reported in the table

***p ≤ 0.01, **0.01 < *p* < 0.05, *0.05 < *p* < 0.1

An encouraging feature for us is that we no longer need to worry about the signs of the pandemic variables in their original forms in the regression. The dependent variable is the log of the quadratic form of the percentage change in stock prices, and a positive or negative sign no longer indicates whether the stock price increases or decreases. Instead, it signals only the size of the change. Specifically, a positive sign only means that the size of the change in stock prices tends to be larger; otherwise, it would be smaller, as a negative sign suggests. Therefore, the marginal effects of all the explanatory variables in this group of models are in the sense of being “larger or smaller in the size of change” (i.e., more or less volatile), rather than having a directional meaning of an increase or decrease in stock prices. This frees us from interpreting the signs of the pandemic variables.

However, according to the theoretical model shown in Eq. (26), the sign of the log of the quadratic form of the difference in the pandemic variable(s) is expected to be positive. In the models presented in Table 5, the variable ICU_patients appears to be problematic, because the sign of the log of the quadratic form of its difference is negative for all the models in this group. For the other four key pandemic variables, daily rates of infection and deaths performed the best, as the sign of the log of the quadratic form of their differences is positive and significant for all the models. The variable of suspected infection is acceptable, as the sign of the corresponding form becomes negative but not significant in two of the models. In addition, the coefficient of the variable for recovery is not statistically significant for all models in this group, although its sign is positive in some of the models. The sign of the log of the stock price variable is still positive in all models, which is inconsistent with our expectations as suggested by the theory.

## Discussion

### Serial correlation versus heteroskedasticity

This study uses a panel data setting; as we have many individual observations (i.e., 3,759 individual stocks) but consider few time periods (i.e., only 16-time points), we might have unbalanced effects concerning the time series and cross-section. In fact, our model extension for the time dimension is very limited, as the fixed and random effects cannot be implemented in the time dimension of our model. Thus, serial correlation over time is not a major concern because the time period is too short to demonstrate a correlation pattern in the series. Therefore, a simple AR(1) term is sufficient to capture the dynamic attributes of the data.

However, heteroskedasticity in the cross-sectional dimension matters in our results. As we have 3,759 individual stocks, representing 3,759 listed companies in 66 different industries, we expect severe heteroskedasticity issues to arise across individuals. As shown in the tables, the cross-sectional estimators tend to have better performance using generalized least squares (GLS) with cross-sectional weights.

### Endogeneity issues

As fluctuations in stock prices, that is, volatility, are very complex, many of the influential factors are unknown. As a result, these omitted variables along with other possible issues may cause endogeneity problems, which lead to inconsistent estimation results. Based on the results in Table 5, we use the variable ICU_patients as the instrumental variable (IV), which is supposed to be uncorrelated with the error term but is correlated with the endogenous variable. We must therefore ask, among all the explanatory variables in our regression, which one is the target endogenous variable? We tried using daily rates of infection, suspected infection, deaths, and recovery, and we chose recovery as the endogenous variable, and the results are shown in Table 6. In fact, it is difficult to coordinate and reconcile the signs for all key pandemic variables using the terms for the log of the quadratic form of their differences. Therefore, we used a combination of recovery and ICU_patients because the results were better in terms of the signs of these terms. Thus, the endogenous variables are recovery as well as ln((∆recovery)^2^), and the corresponding IVs are ICU_patients as well as log((∆ICU_patients)^2^). The difference in J-statistics for the endogeneity test is 287.848 with a *p*-value of 0.000, and the Cragg-Donald F-stat for the weak instrument diagnostics is 1,576.949. Therefore, we feel justified using this combination.

**Table 6:** Empirical estimation results with dependent variable ln((Pct_chg)^2^) of pooled or panel least square with or without AR(1) term with complete theoretical guidance with IVs (*n* = 60,144)

	Model (6–1) (Pooled IV/two-stage least squares)	Model (6–2) (Pooled IV /two-stage EGLS with cross-section random effects)	Model (6–3) (Pooled IV/two-stage EGLS with cross− section weights)	Model (6–4) (Pooled IV/two-Stage EGLS with period weights)	Model (6–5) (Pooled IV/two-stage EGLS with period SUR)	Model (6–6) (Pooled IV/two-stage least squares with AR(1))	Model (6–7) (Pooled IV/two-stage least squares with cross-section weights and AR(1))
ln((Close)^2^)	0.238*** (25.752)	0.261*** (21.266)	0.170*** (24.641)	0.176*** (20.622)	0.168*** (16.341)	0.247*** (24.815)	0.170*** (23.403)
Daily_infection	− 0.001*** (− 11.099)	− 0.001*** (− 11.596)	− 0.001*** (− 15.382)	− 0.000** (− 10.233)	− 0.000*** (− 11.445)	0.000*** (6.268)	0.000*** (9.059)
ln(∆Daily_infection^2^)	− 0.049 (− 1.607)	− 0.049 (− 1.679)	− 0.030 (− 1.396)	− 0.118*** (− 4.014)	− 0.087*** (− 3.041)	− 0.128*** (− 4.698)	− 0.109*** (− 5.949)
Suspected_infection	− 0.001*** (− 25.497)	− 0.001*** (− 26.649)	− 0.001*** (− 33.982)	− 0.000*** (− 31.875)	− 0.000*** (− 32.101)	− 0.000*** (− 21.222)	− 0.000*** (− 28.236)
ln((∆Suspected_infection_new)^2^)	0.023*** (5.366)	0.023*** (5.607)	0.025*** (8.008)	0.018*** (4.813)	0.022*** (6.014)	− 0.028*** (− 11.461)	− 0.026*** (− 16.043)
Death_cases	0.025*** (8.570)	0.025*** (8.953)	0.025*** (12.122)	0.019*** (7.499)	0.021*** (6.014)	− 0.013*** (− 13.659)	− 0.012*** (− 19.027)
ln((∆Death_cases)^2^)	0.135 (1.409)	0.136 (1.486)	0.018 (0.262)	0.467*** (5.318)	0.309*** (3.362)	0.855*** (16.147)	0.718*** (20.049)
Recovery	− 0.002*** (− 9.936)	− 0.002*** (− 10.381)	− 0.001*** (− 13.889)	− 0.001*** (− 9.235)	− 0.001*** (− 10.427)	0.000*** (9.665)	0.000*** (13.544)
ln((∆Recovery)^2^)	0.602*** (8.469)	0.601*** (8.830)	0.606*** (12.039)	0.318*** (5.532)	0.423*** (7.214)	0.094*** (2.641)	0.129*** (5.360)
Total_revenue	− 1.00E−08** (− 2.050)	− 1.04E−08 (− 1.597)	− 8.52E−09** (− 2.077)	− 1.02E−08** (− 2.268)	− 9.46E−09* (− 1.736)	− 6.22E−09 (− 1.182)	− 5.32E−09 (− 1.544)
ln(Operating_cost)	− 0.108*** (− 10.218)	− 0.109*** (− 7.835)	− 0.102*** (− 13.491)	− 0.112*** (− 11.496)	− 0.108*** (− 9.172)	− 0.101*** (− 8.946)	− 0.094*** (− 12.116)
Operating_profit	− 7.30E−08*** (− 3.707)	− 7.48E−08*** (− 2.853)	− 9.08E−08*** (− 4.248)	− 7.03E−08*** (− 3.869)	− 6.89E−08*** (− 3.135)	− 8.94E−08*** (− 4.211)	− 1.08E−07** (− 4.964)
ln(Buy_vol)	0.149*** (18.886)	0.159*** (15.229)	0.146*** (24.862)	0.123*** (16.973)	0.122*** (13.946)	0.157*** (18.530)	0.151*** (24.766)
Industry_5	0.655*** (7.415)	0.656*** (5.579)	0.542*** (8.826)	0.561*** (6.881)	0.505*** (5.128)	0.668*** (7.014)	0.571*** (8.907)
Industry_7	0.415*** (3.797)	0.404*** (2.779)	0.348*** (4.559)	0.344*** (3.408)	0.366*** (3.001)	0.465*** (3.946)	0.392*** (4.897)
Industry_8	− 0.790*** (− 2.958)	− 0.757** (− 2.128)	− 0.376 (− 1.283)	− 0.541** (− 2.194)	− 0.491* (− 1.649)	− 0.931*** (− 3.232)	− 0.455 (− 1.447)
Industry_9	− 1.509*** (− 9.162)	− 1.481*** (− 6.747)	− 1.362*** (− 8.133)	− 1.618*** (− 10.642)	− 1.619*** (− 8.804)	− 1.443*** (− 8.124)	− 1.213*** (− 7.469)
Industry_10	0.588*** (4.107)	0.568*** (2.977)	0.469*** (4.708)	0.492*** (3.724)	0.479*** (2.995)	0.612*** (3.963)	0.519*** (4.829)
Industry_15	0.176** (2.159)	0.175 (1.607)	0.069 (1.174)	0.161** (2.137)	0.133 (1.461)	0.209** (2.382)	0.102 (1.634)
Industry_16	0.223** (2.312)	0.206 (1.604)	0.131* (1.831)	0.049 (0.555)	− 0.065 (− 0.601)	0.247** (2.215)	0.181** (2.402)
Industry_19	0.235** (2.268)	0.203 (1.476)	0.215*** (2.868)	0.188** (1.967)	0.181 (1.571)	0.247** (2.215)	0.261*** (3.222)
Industry_21	0.519*** (4.904)	0.469*** (3.328)	0.486*** (6.410)	0.374*** (3.829)	0.367*** (3.107)	0.569*** (4.992)	0.576*** (7.044)
Industry_22	− 0.627*** (− 2.692)	− 0.624** (− 2.012)	− 1.013*** (− 5.762)	− 0.486** (− 2.262)	− 0.327 (− 1.258)	− 0.494*** (− 2.764)	− 1.069*** (− 6.024)
Industry_23	0.265** (2.327)	0.246 (1.625)	0.125 1.559	0.359*** (3.385)	0.385*** (3.029)	0.279** (2.269)	0.145* (1.678)
Industry_26	− 0.205* (− 1.824)	− 0.197 (− 1.316)	− 0.380*** (− 4.865)	− 0.304*** (− 2.940)	− 0.319** (− 2.551)	− 0.189 (− 1.571)	− 0.370*** (− 4.519)
Industry_27	− 0.588*** (− 6.297)	− 0.569*** (− 4.580)	− 0.613*** (− 8.940)	− 0.563*** (− 6.541)	− 0.515*** (− 4.942)	− 0.589*** (− 5.864)	− 0.577*** (− 8.179)
Industry_28	− 0.711*** (− 7.474)	− 0.687*** (− 5.419)	− 0.656*** (− 8.629)	− 0.823*** (− 9.376)	− 0.778*** (− 7.329)	− 0.718*** (− 6.994)	− 0.629*** (− 7.866)
Industry_29	0.577*** (4.036)	0.565*** (2.968)	0.423** (4.615)	0.516*** (3.908)	0.460** (2.886)	0.618*** (− 6.994)	0.489*** (4.782)
Industry_34	0.332* (1.928)	0.357 (1.554)	0.202* (1.677)	0.281* (1.765)	0.347** (1.804)	0.381** (2.044)	0.248** (2.004)
Industry_36	− 1.034*** (− 6.550)	− 1.006*** (− 4.787)	− 0.776*** (− 6.350)	− 0.795*** (− 5.458)	− 0.542*** (− 3.078)	− 1.124*** (− 6.604)	− 0.822*** (− 6.333)
Industry_37	− 0.754*** (− 5.139)	− 0.728*** (− 3.724)	− 0.883*** (− 8.155)	− 0.769*** (− 5.681)	− 0.733*** (− 4.475)	− 0.678*** (− 4.286)	− 0.774*** (− 7.489)
Industry_38	− 0.783*** (− 5.764)	− 0.766*** (− 4.232)	− 0.901** (− 9.129)	− 0.723** (− 5.769)	− 0.659*** (− 4.347)	− 0.813*** (− 5.547)	− 0.903*** (− 9.330)
Industry_41	0.392*** (2.703)	0.364* (1.884)	0.272*** (2.709)	0.191 (1.425)	0.048 (0.295)	0.432*** (2.754)	0.345*** (3.348)
Industry_42	− 1.001*** (− 9.028)	− 0.975*** (− 6.602)	− 0.814*** (− 9.052)	− 0.958*** (− 9.366)	− 0.879*** (− 7.107)	− 0.956*** (− 7.998)	− 0.773*** (− 8.474)
Industry_43	0.324*** (2.775)	0.289* (1.857)	0.234*** (2.922)	0.329*** (3.058)	0.399*** (3.062)	0.347*** (2.759)	0.299*** (3.571)
Industry_46	− 0.935*** (− 3.841)	− 0.924** (− 2.851)	− 0.609** (− 2.946)	− 0.899*** (− 4.004)	− 0.821*** (− 3.021)	− 0.882*** (− 3.359)	− 0.569*** (− 2.731)
Industry_47	0.556*** (3.076)	0.558** (2.320)	0.459*** (3.854)	0.541*** (3.245)	0.552*** (2.737)	0.625*** (3.211)	0.540*** (4.196)
Industry_50	0.566** (2.325)	0.575* (1.774)	0.506*** (2.670)	0.604*** (2.693)	0.590** (2.174)	0.547** (2.085)	0.533*** (2.726)
Industry_51	0.687*** (8.213)	0.656*** (5.886)	0.641*** (10.969)	0.583*** (7.552)	0.567*** (6.065)	0.739*** (8.199)	0.729*** (11.794)
Industry_52	0.840*** (6.422)	0.821*** (4.712)	0.585*** (6.785)	0.705*** (5.839)	0.677*** (4.635)	0.889*** (6.299)	0.649*** (7.145)
Industry_53	− 0.525*** (− 3.839)	− 0.541*** (− 2.975)	− 0.547*** (− 4.996)	− 0.356*** (− 2.826)	− 0.274* (− 1.795)	− 0.456*** (− 3.098)	− 0.447*** (− 3.849)
Industry_54	− 0.497*** (− 2.903)	− 0.467** (− 2.051)	− 0.398*** (− 2.729)	− 0.482*** (− 3.057)	− 0.408** (− 2.138)	− 0.523*** (− 2.837)	− 0.372** (− 2.438)
Industry_55	0.588*** (4.011)	0.567*** (2.901)	0.614*** (5.891)	0.509*** (3.760)	0.482*** (2.945)	0.667*** (4.218)	0.700*** (6.479)
Industry_56	0.598*** (6.081)	0.578*** (4.413)	0.450*** (6.530)	0.555*** (6.115)	0.599*** (5.465)	0.609*** (5.738)	0.483*** (6.562)
Industry_58	− 0.574*** (− 3.105)	− 0.556** (− 2.258)	− 0.574*** (− 3.731)	− 0.484*** (− 2.838)	− 0.371* (− 1.797)	− 0.558*** (− 2.801)	− 0.537*** (− 3.304)
Industry_60	− 0.416** (− 2.041)	− 0.399 (− 1.467)	− 0.434*** (− 2.687)	− 0.360* (− 1.912)	− 0.294 (− 1.293)	− 0.479** (− 2.181)	− 0.497*** (− 2.933)
Industry_61	− 0.612*** (− 4.036)	− 0.585*** (− 2.895)	− 0.594*** (− 5.574)	− 0.645*** (− 4.612)	− 0.560*** (− 3.311)	− 0.590*** (− 3.609)	− 0.595*** (− 5.312)
Industry_62	− 1.003*** (− 6.226)	− 0.988*** (− 4.604)	− 1.139*** (− 10.934)	− 0.925*** (− 6.221)	− 0.803*** (− 4.466)	− 1.018*** (− 5.858)	− 1.162*** (− 10.686)
Industry_63	− 0.351*** (− 3.217)	− 0.329** (− 2.271)	− 0.387*** (− 4.694)	− 0.302*** (− 3.002)	− 0.245** (− 2.018)	− 0.358*** (− 3.050)	− 0.367*** (− 4.366)
Industry_66	− 1.125*** (− 8.275)	− 1.138*** (− 6.285)	− 0.998*** (− 9.351)	− 1.074*** (− 8.559)	− 1.003*** (− 6.612)	− 1.120*** (− 7.645)	0.054*** (12.579)
AR(1)						0.045*** (10.213)	0.054*** (12.579)
Random effect (cross)		Yes					
Adjusted *R*^2^	0.082	0.061	0.117	0.112	0.090	0.101	0.152

The values of the constant terms are not reported. *t* statistics in parentheses. Instrumental variables (IVs) are ICU_patients as well as log((∆ICU_patients)^2^). For industrial variables, only those who are statistically significant are reported in the table

***p ≤ 0.01, **0.01 < *p* < 0.05, *0.05 < *p* < 0.1

The results in Table 6 are generally similar to those listed in Table 5. However, after the endogeneity problem is addressed, the sign of the log of the quadratic form of its difference for many of the key variables is now positive and significant. Although it is difficult to have all of these terms to be positive and significant, Model (6–3) appears to be our best shot, because the term for daily rates of infection with the log of the quadratic form of its difference is not significant even though its sign remains negative. In comparison, although Model (6–7) has the largest adjusted *R*^*2*^, this term for daily rates of infection is negative and significant, which is not the desired result based on the theoretical framework proposed earlier.

### Pandemic or panic?

Considering the title of this paper, we pose the question of whether the COVID-19 pandemic causes panic in the stock market. Our empirical results indicate that the answer is yes. Tables 3 and 4 show the ideal results for the pandemic variables, in which daily rates of infection, suspected infection, deaths, and ICU patients should have negative and significant coefficients. This means that when there are more reported data on these four variables, there is greater panic in the stock market, and hence, the daily returns of the stock prices tend to be lower. In contrast, the variable for recovery is expected to have a positive and significant coefficient. Although the empirical results are difficult to reconcile with the theoretical setup, we attempt to improve the models.

When we enhance the empirical models using the theoretical framework, the outlook becomes more interesting. As mentioned earlier, when the log of the quadratic form is used for the percentage change in stock prices as the dependent variable, we reveal another hidden mechanism in investor reactions to the pandemic in the sense that we have quantified the degree of the reaction in the stock market. Although the direction of price change is no longer our concern, the size of change does matter, and we confirm this in our empirical results shown in Tables 5 and 6.

As mentioned earlier, most studies related to “shocks” to the financial market are empirical. Although methods such as impulse function can capture the facts and features of this relationship, they cannot explain how and why. For example, when we throw a pebble into a pool, it becomes a “black swan” event; then, when the stone hits the water, it causes ripples, which become the impulses in the empirical models. However, empirical models cannot explain the reason for or the method of the ripples.

To our knowledge, although “shocks” to the financial market are mainly discussed based on empirical evidence, the real mechanism in shocks is still unknown. Equations (24) and (26) in our theoretical model, however, show the mechanism of the shock. The terms $((Psy^{\prime\prime}(Epi) + Ind^{\prime\prime}(Epi))Ind^{\prime\prime}(Epi))Epi + \ln (\mathop {Epi}\limits^{ \bullet } )$ in Eq. (24), as well as the $2((Psy^{\prime\prime}(Epi) + Ind^{\prime\prime}(Epi))Ind^{\prime\prime}(Epi))Epi + \ln ((\mathop {Epi}\limits^{ \bullet } )^{2} )$ in Eq. (26) are essentially the “ripples,” as they offer novel insights into not only why “black swan” events, such as the COVID-19 pandemic, cause shocks to stock prices but also the specific functional expression for calculating the shock.

In addition, Table 6 has a more interesting feature as well; the absolute values of the log of the quadratic form of the differences in the pandemic variables are much larger than those in their original forms, which means that the terms for these incremental differences eventually dominate the marginal effect. If this finding is connected to the impulse mentioned earlier, it confirms the impulse reaction of the shock fades. For example, we look at the variable recovery in Model (6–3); its coefficient is -0.001, whereas the coefficient of ln((∆recovery)^2^) is 0.606. The absolute value of the latter is much larger (i.e., approximately 600 times larger) than that of the former (i.e., the cumulative level). Therefore, as the infection rate stabilizes, the daily difference diminishes, resulting in a smaller marginal influence on changes in stock prices. Hence, the impulse fades to the point of disappearance.

### Do fundamentals matter?

Although this study uses stock fundamentals as control variables, which are not our primary research targets, it confirms several interesting facts. In Model (6–2), for example, the sign of total revenue is negative, which means that smaller companies have more volatile stock prices. This principle also applies to operating cost and operating profit. Together, these results confirm that the scale of listed firms has a negative impact on fluctuations in their stock prices, which is reasonable.

In addition, the positive and significant sign of the buying volume is meaningful in the sense that it identifies the demand side of the stock market, in which more buying volume raises stock prices as well as the size of changes. This result confirms the standard laws of supply and demand, even in a more uncertain market, such as the stock market.

### Revisiting the industry variables

**Fig. 3:**
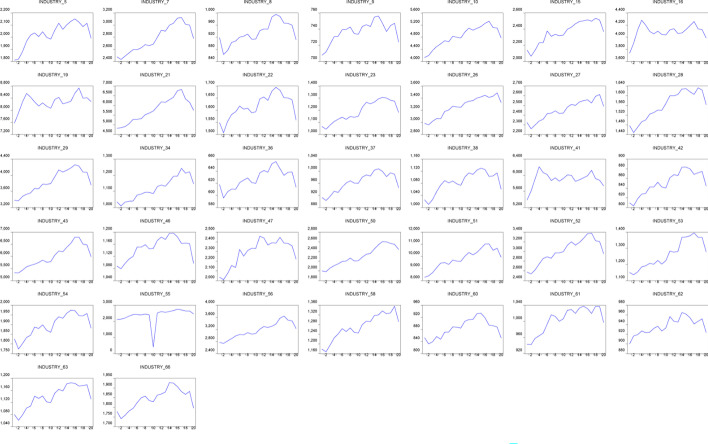
The trend in industrial indices for selected industries in the Chinese stock market in February 2020. *Note*: In all the figures, the x-axis is the period from February 3 to 28, 2020, and the y-axis comprises various industrial indices

An important feature of this study, other than the group of control variables, is the use of industry dummy variables. We are particularly interested in the diverse responses of different industries in confronting the COVID-19 pandemic. Figure 3 shows the trend in industrial indices for selected industries in the Chinese stock market in February 2020. Of a total of 66, these 37 industries are selected based on the significance of the dummy variables shown in Table 6; they show substantial differences in the trend of indices among industries, which confirms the “belief dispersion” commonly seen in the stock market (Atmaz and Basak 2018).

A clear upward trend is seen after the stock market crash on February 3, for instance, in industry_7 (optics and optoelectronics), industry_10 (other electronics), and industry_21 (semiconductor and its components). However, a sharp drop is found in other industries, such as industry_8 (public transport), industry_22 (park development), and industry_55 (communication service). Moreover, in other industries, including industry_16 (chemical pharmacy), industry_19 (medical equipment services), and industry_41 (biological products), the trends remained relatively stable after an initial sharp rise after February 3.

All of these results are common. As the “black swan” event in this study is due to a pandemic, health-related industries experienced a rapid rise after the stock market in China reopened, and their indices remained relatively high. Notably, these industries directly benefited from the pandemic. In addition, many other industries, especially manufacturing, gradually increased, as recovery from the overreaction on the first day that the stock market reopened. However, industries such as public transport, park development, and communications services were directly affected by the pandemic, so investors’ panic caused a sharp drop in stock prices. The results also demonstrate the revival of businesses that needed to return to normal during the COVID-19 pandemic (Brammer et al. 2020).

## Concluding remarks

This study investigates the relationship between the COVID-19 pandemic and daily changes in stock prices in China in February 2020 as an example. It presents the impact mechanism for the effect of the COVID-19 pandemic on the stock market by pointing out the specific functional expression of the impulse reaction. The impact mechanism was further analyzed based on theoretical and empirical evidence. The research also empirically estimates the marginal effect of the pandemic on stock returns. The results show that the shock from the COVID-19 pandemic to the market is heterogeneous across industries, and the panic caused by the pandemic expands volatility in daily returns, but the impulse of the shock from the pandemic eventually fades.

Stock market prices are important indicators of the rise and fall in the direction of the economy, and their movements also drive economic fluctuations. Therefore, this study provides a theoretical model for exploring the quantitative relationship between COVID-19 and stock prices and empirically tests the relationship using China’s stock market as an experimental laboratory. Most extant studies on similar topics are empirical, whereas this study offers new insights into the mechanism through which the pandemic affects the stock market with a specific functional expression. In addition, this study conducts a comprehensive examination through various regressions and indicators, and the results have important policy implications.

In the theoretical framework, this study considers three types of influential factors—fundamental factors, psychological factors, and industrial factors—of which the psychological and industrial impacts of COVID-19 on the stock market are of particular interest. In the empirical section, based on a Chinese sample, this study focuses on February 3–25, 2020, a period during which it is possible to isolate the effect of the COVID-19 pandemic on local stock performance.

The study has several interesting findings.

First, in terms of stock fundamentals, the study finds that total revenue is negative, whereas buying volume is positively correlated with stock prices.

Second, as confirmed in this study, the COVID-19 pandemic creates panic in the stock market, which not only depresses stock prices but also enhances volatility in daily returns. Concerning the impulse of the shock, we identify both the cumulative level of the pandemic variables and their incremental differences. Our empirical results show that the terms for these incremental differences eventually dominate the marginal effect, which confirms that the impulse of the shock fades out.

Third, regarding the heterogeneous response across industries, almost every industry overreacted on February 3, 2020, when the stock markets in China reopened after the national holiday break. This mass overreaction caused a stock market crash as a result of a “black swan” event. However, after the crash, industries that directly benefited from the pandemic increased rapidly, whereas those that were negatively affected by the pandemic continued to drop. Moreover, many other industries that were harmed by panic due to the pandemic recovered, which is typical of the impulse response to a black swan event in the financial market.

This study also offers several important policy implications based on theoretical and empirical findings.

First, regulatory policymakers need to take a closer look at the daily caseload (i.e., the daily increase in infection), not just the cumulative level. If daily changes in the key pandemic indices are increasing, policymakers should be aware of its negative shock potential towards the stock market. Thus, appropriate policies need to be implemented or announced to offset adverse shocks to the stock market. Otherwise, if such daily changes are decreasing, it means that the impact of the pandemic on the stock market is fading out, the implication of which is that the regulators do not need to worry too much about the fluctuation in the stock market.

Second, as the pandemic winds down in some regions, returning to work becomes the most important factor for local communities. Appropriate stimulation policies could be implemented, especially for industries that suffered significant losses because of the pandemic. As mentioned earlier, some industries overreact and recover to some degree. However, for some other industries, the impact of the pandemic is long-term, so they need assistance from the government to recover fully.

Third, this study offers guidance to practitioners, such as stock market investors, especially those who are more interested in industries rather than individual stocks. It provides a comprehensive industrial analysis of all economic sectors covered by the Chinese stock markets. The heterogeneous responses to a “black swan” event in the different industries examined in this study thus have high practical value.

This study should have a broad interest. Although the topics studied in this paper are the COVID-19 pandemic and the Chinese stock market, they can be replaced by black swan events of any kind as well as the financial markets in any region. In addition to the COVID-19 pandemic, there are many other types of shocks to the stock market. Each time, some industries are vulnerable, while others are robust. Therefore, we hope that the methodological contribution, as well as the insights of this study, can be sufficiently general.

Nevertheless, some issues remain that we could not address at the current stage of our research. First, the time effect on stock prices and the performance of listed companies must be taken into account. The stock prices we obtained cover less than a one-month period, so having more information would help us identify the effects of COVID-19 on changes in stock prices more accurately. Second, to obtain more precise results, future studies also need to include samples from countries other than China, as they might have some correlation with stock prices in China. Most prominently, on February 26, 2020, when the US stock markets and global financial markets began to fall, the stock market in China turned down again. We leave this part of the story for future studies.

Finally, although it would be very meaningful to consider controlling for regional effects, such as the province or city effect, we do not do so in this study for two main reasons. First, many listed companies have their headquarters in one place (i.e., province or city), but their business operations may be distributed across many other locations. This makes it very difficult to decide which location should be counted. Second, individual investors in a particular stock can be anywhere because of internet-based transactions. Therefore, the location of individual investors may be irrelevant. In fact, some empirical estimation results are inconsistent with the signs suggested by the expectations of our theory. If more control variables, such as regional factors, are included, the empirical estimation results might fare better at the risk of introducing other unexpected problems. For these reasons, although we did not include these control variables in this study, we would consider doing so in a future study.

## Data Availability

All data used in this study are publicly available.

## Appendix

Here, we begin with Eq. (19), shown earlier. If C_4_ = 0, then the calculation is much easier. Because C_4_ is expressed as the subtraction of two integration constants, then the zero assumption here is reasonable. Now, we have:

28 $$Psy(Epi) + Ind(Epi) = \frac{{\exp ((Ind^{\prime}(Epi) - C_{1} )(Psy^{\prime\prime}(Epi) + Ind^{\prime\prime}(Epi)))}}{{(Psy^{\prime\prime}(Epi) + Ind^{\prime\prime}(Epi))Ind^{\prime\prime}(Epi)}}.$$

Taking the log form on both sides of the equation, we have:

29 $$\begin{aligned} & \ln (Psy(Epi) + Ind(Epi)) + \ln (Psy^{\prime\prime}(Epi) + Ind^{\prime\prime}(Epi)) + \ln (Ind^{\prime\prime}(Epi)) \\ & \quad= (Psy^{\prime\prime}(Epi) + Ind^{\prime\prime}(Epi))\ln ((\exp (Ind^{\prime}(Epi) - C_{1} )). \\ \end{aligned}$$

Now, we integrate the equation on both sides, and we have:

30 $$\begin{aligned} & \ln (Psy(Epi) + Ind(Epi)) + \ln (Psy^{\prime\prime}(Epi)+ Ind^{\prime\prime}(Epi)) + \ln (Ind^{\prime\prime}(Epi))\\ & \quad = (Psy^{\prime\prime}(Epi) + Ind^{\prime\prime}(Epi))\ln ((\exp (Ind^{\prime}(Epi) - C_{1} )). \\ \end{aligned}$$

On the left-hand side of the equation, we integrate it by parts and obtain:

31 $$\begin{aligned} & \int {\ln ((Psy(Epi) + Ind(Epi))(Psy^{\prime\prime}(Epi)} + Ind^{\prime\prime}(Epi))Ind^{\prime\prime}(Epi))dEpi \\ & \quad = Epi\ln ((Psy(Epi) + Ind(Epi))(Psy^{\prime\prime}(Epi) + Ind^{\prime\prime}(Epi))Ind^{\prime\prime}(Epi)) \\ & \quad \quad - \int {Epid} \ln ((Psy(Epi) + Ind(Epi))(Psy^{\prime\prime}(Epi) + Ind^{\prime\prime}(Epi))Ind^{\prime\prime}(Epi)). \\ \end{aligned}$$

Noting that,

32 $$\begin{aligned} & \int {Epid} \ln ((Psy(Epi) + Ind(Epi))(Psy^{\prime\prime}(Epi) + Ind^{\prime\prime}(Epi))Ind^{\prime\prime}(Epi)) \\ & \quad = \int {Epi\frac{{(Psy^{\prime}(Epi) + Ind^{\prime}(Epi))(Psy^{\prime\prime}(Epi) + Ind^{\prime\prime}(Epi))Ind^{\prime\prime}(Epi)}}{{(Psy(Epi) + Ind(Epi))(Psy^{\prime\prime}(Epi) + Ind^{\prime\prime}(Epi))Ind^{\prime\prime}(Epi)}}d} Epi \\ & \quad = \int {Epi\frac{{(Psy^{\prime}(Epi) + Ind^{\prime}(Epi))}}{(Psy(Epi) + Ind(Epi))}d} Epi \\ & \quad = \int {Epid} \ln (Psy(Epi) + Ind(Epi)). \\ \end{aligned}$$

Now we do it again, using the integration by parts of Eq. 32:

33 $$\begin{aligned} \int {Epid} \ln (Psy(Epi) + Ind(Epi))&= Epi\ln (Psy(Epi) + Ind(Epi)) \\ & \quad - \int {\ln (Psy(Epi) + Ind(Epi))} dEpi. \\ \end{aligned}$$

Substituting Eq. (33) into Eq. (31), we have:

34 $$\begin{aligned} & \int {\ln ((Psy(Epi) + Ind(Epi))(Psy^{\prime\prime}(Epi) + Ind^{\prime\prime}(Epi))Ind^{\prime\prime}(Epi))} dEpi \\ & \quad = Epi\ln (\frac{{(Psy(Epi) + Ind(Epi))(Psy^{\prime\prime}(Epi) + Ind^{\prime\prime}(Epi))Ind^{\prime\prime}(Epi)}}{Psy(Epi) + Ind(Epi)}) \\ & \quad\quad + \int {\ln (Psy(Epi) + Ind(Epi))} dEpi \\ & \quad = Epi\ln ((Psy^{\prime\prime}(Epi) + Ind^{\prime\prime}(Epi))Ind^{\prime\prime}(Epi)) + \int {\ln (Psy(Epi) + Ind(Epi))} dEpi. \\ \end{aligned}$$

The integration on the right-hand side of Eq. (30) is relatively simple, hence we have:

35 $$\begin{aligned} & \int {(Psy^{\prime\prime}(Epi) + Ind^{\prime\prime}(Epi))(Ind^{\prime}(Epi) - C_{1} )} dEpi \\ & \quad = (Psy^{\prime\prime}(Epi) + Ind^{\prime\prime}(Epi))(Ind(Epi) - C_{1} ) + C_{5} \\ & \quad = (Psy^{\prime\prime}(Epi) + Ind^{\prime\prime}(Epi))Ind(Epi) + C_{6} . \\ \end{aligned}$$

where $$C_{{6}} { = }C_{5} - (Psy^{\prime\prime}(Epi) + Ind^{\prime\prime}(Epi))C_{1}$$.

If we combine the left- and the right-hand sides of Eq. (30), we have:

36 $$\begin{aligned} & Epi\ln ((Psy^{\prime\prime}(Epi) + Ind^{\prime\prime}(Epi))Ind^{\prime\prime}(Epi)) \\ & \quad + \int {\ln (Psy(Epi) + Ind(Epi))} dEpi \\ & \quad = (Psy^{\prime\prime}(Epi) + Ind^{\prime\prime}(Epi))Ind(Epi) + C_{6} . \\ \end{aligned}$$

After rearrangement, we have:

37 $$\begin{aligned} Ind(Epi) & = \frac{{\ln ((Psy^{\prime\prime}(Epi) + Ind^{\prime\prime}(Epi))Ind^{\prime\prime}(Epi))}}{{Psy^{\prime\prime}(Epi) + Ind^{\prime\prime}(Epi)}}Epi \\ & \quad + \frac{{\int {\ln (Psy(Epi) + Ind(Epi))} dEpi}}{{Psy^{\prime\prime}(Epi) + Ind^{\prime\prime}(Epi)}} \\ & \quad - \frac{{C_{6} }}{{Psy^{\prime\prime}(Epi) + Ind^{\prime\prime}(Epi)}}. \\ \end{aligned}$$

Now, treating all the second-order terms as constants, we differentiate the equation on both sides, and we have:

38 $$\begin{aligned} Ind^{\prime}(Epi) & = \frac{{\ln ((Psy^{\prime\prime}(Epi) + Ind^{\prime\prime}(Epi))Ind^{\prime\prime}(Epi))}}{{Psy^{\prime\prime}(Epi) + Ind^{\prime\prime}(Epi)}} \\ & \quad + \frac{\ln (Psy(Epi) + Ind(Epi))}{{Psy^{\prime\prime}(Epi) + Ind^{\prime\prime}(Epi)}}. \\ \end{aligned}$$

After rearrangement, we have:

39 $$\begin{aligned} & (Psy^{\prime\prime}(Epi) + Ind^{\prime\prime}(Epi))Ind^{\prime}(Epi) \\ & \quad = \ln ((Psy^{\prime\prime}(Epi) + Ind^{\prime\prime}(Epi))Ind^{\prime\prime}(Epi)(Psy(Epi) + Ind(Epi))). \\ \end{aligned}$$

Now, still treating all the second-order terms as constants, we differentiate the equation on both sides again, and we have:

40 $$\begin{aligned} & (Psy^{\prime\prime}(Epi) + Ind^{\prime\prime}(Epi))Ind^{\prime\prime}(Epi) \\ & \quad = \frac{{(Psy^{\prime\prime}(Epi) + Ind^{\prime\prime}(Epi))Ind^{\prime\prime}(Epi)(Psy^{\prime}(Epi) + Ind^{\prime}(Epi))}}{{(Psy^{\prime\prime}(Epi) + Ind^{\prime\prime}(Epi))Ind^{\prime\prime}(Epi)(Psy(Epi) + Ind(Epi))}} \\ & \quad = \frac{{(Psy^{\prime}(Epi) + Ind^{\prime}(Epi))}}{(Psy(Epi) + Ind(Epi))}. \\ \end{aligned}$$

Now for the last time, we integrate both sides of the equation and obtain:

41 $$\begin{aligned} & \int {((Psy^{\prime\prime}(Epi) + Ind^{\prime\prime}(Epi))Ind^{\prime\prime}(Epi))d} Epi \\ & \quad = \int {\frac{{(Psy^{\prime}(Epi) + Ind^{\prime}(Epi))}}{(Psy(Epi) + Ind(Epi))}} dEpi. \\ \end{aligned}$$

Then,

42 $$\begin{aligned} ((Psy^{\prime\prime}(Epi) + Ind^{\prime\prime}(Epi))Ind^{\prime\prime}(Epi))Epi + C_{7} = \ln (Psy(Epi) + Ind(Epi)) + C_{8} . \\ \end{aligned}$$

which is,

43 $$\begin{aligned} \ln (Psy(Epi) + Ind(Epi)) = ((Psy^{\prime\prime}(Epi)+ Ind^{\prime\prime}(Epi))Ind^{\prime\prime}(Epi))Epi + C_{{9}} . \\ \end{aligned}$$

whsere $$C_{{9}} = C_{7} - C_{8}$$.

Finally, letting C_9_ be zero and taking the exponential form on both sides of the equation, we obtain:

44 $$\begin{aligned} Psy(Epi) + Ind(Epi) & = \exp (((Psy^{\prime\prime}(Epi) + Ind^{\prime\prime}(Epi))Ind^{\prime\prime}(Epi))Epi) \\ & = (\exp (Epi))^{{((Psy^{\prime\prime}(Epi) + Ind^{\prime\prime}(Epi))Ind^{\prime\prime}(Epi))}} . \\ \end{aligned}$$

## Footnotes

1 World Health Organization (WHO), https://www.who.int.

2 China Securities Regulatory Commission (CSRC). http://www.csrc.gov.cn/pub/newsite.

3 Global Rubber Markets: https://globalrubbermarkets.com/195006/china-virus-outbreak-pressures-already-weakened-economy.html.

4 Ministry of Transport of the People’s Republic of China, http://www.mot.gov.cn/shuju/.

5 Shanghai Jiao Tong University Industry Research Institute. The impacts of COVID-19 on several industries in China. February, 2020. http://www.iir.sjtu.edu.cn/news/42488.html.

6 China Cuisine Association. The impacts of COVID-19 on China's catering industry in 2020.http://www.ccas.com.cn/site/content/204393.html?siteid=1/.

7 USA TODAY, https://www.usatoday.com/story/money/2020/01/26/coronavirus-hits-stock-markets-united-airlines-travel-stocks-drop/4581568002/.

8 Nasdaq < Coronavirus Could Trigger More Stock Selloffs. Look for a Buying Opportunity, J.P. Morgan says > , https://www.nasdaq.com/articles/coronavirus-could-trigger-more-stock-selloffs.-look-for-a-buying-opportunity-j.p.-morgan/.

9 Nasdaq < Coronavirus Could Trigger More Stock Selloffs. Look for a Buying Opportunity, J.P. Morgan says > , https://www.nasdaq.com/articles/coronavirus-could-trigger-more-stock-selloffs.-look-for-a-buying-opportunity-j.p.-morgan/.

10 Economic Times < Indian stock markets not under much pressure over China virus > , https://economictimes.indiatimes.com/markets/stocks/news/indian-stock-markets-not-under-much-pressure-over-china-virus/articleshow/73650172.cms.

11 The State Council Information Office of the People’s Republic of China, http://www.scio.gov.cn/ztk/dtzt/37868/38521/38531/Document/1632373/1632373.htm.

12 China Economic Times, http://lib.cet.com.cn/paper/szb_con/108414.html.

13 China Daily, http://www.chinadaily.com.cn/en/doc/2003-10/17/content_273199.htm.

14 BBC news, http://www.bbc.com/news/uk-politics-32810887/.

15 Indian Express news, https://indianexpress.com/article/world/italy-pm-renzi-to-resign-after-defeat-on-constitutional-referendum-euro-falls-to-20-month-low-4411655/.

16 National Health Commission of the People’s Republic of China, http://www.nhc.gov.cn.

17 East Money, https://www.eastmoney.com.
